# Interleukin 35-producing B cells prolong the survival of GVHD mice by secreting exosomes with membrane-bound IL-35 and upregulating PD-1/LAG-3 checkpoint proteins

**DOI:** 10.7150/thno.105069

**Published:** 2025-02-25

**Authors:** Jin Kyeong Choi, Evaristus C. Mbanefo, Manoj Kumar Yadav, Sahar A. Alhakeem, Vijayaraj Nagarajan, Natalia S. Nunes, Christopher G. Kanakry, Charles E. Egwuagu

**Affiliations:** 1 Molecular Immunology Section, Laboratory of Immunology, National Eye Institute (NEI), National Institutes of Health (NIH), Bethesda, MD 20892, United States of America (USA).; 2 Department of Immunology, Jeonbuk National University Medical School, Jeonju, Jeonbuk, 54907, Republic of Korea.; 3 Department of Biomedical Sciences, College of Health Sciences, University of Wisconsin-Milwaukee, Milwaukee, WI, United States; 4 Center for Immuno-Oncology, Center for Cancer Research, National Cancer Institute, National Institutes of Health, Bethesda MD 20892, United States of America.

**Keywords:** interleukin-35, Bregs, exosomes, immunotherapy, immune checkpoint proteins, infectious tolerance, transplantation, GVHD

## Abstract

**Background:** Allogeneic hematopoietic stem cell transplantation (allo-HSCT) is an effective treatment for aggressive hematologic malignancies. However, the risk of developing graft-versus-host disease (GVHD) is a significant barrier to allo-HSCT. GVHD is a debilitating condition with high mortality rates and current therapeutic options for GVHD are limited, with corticosteroids being the standard treatment. However, the adverse effects of steroids make prolonged use difficult, necessitating the development of safer therapies. IL-35-producing B-cells (i35-Bregs) have emerged as critical regulators of immunity during autoimmune diseases. In this study, we investigated whether i35-Bregs immunotherapy can suppress and mitigate GVHD.

**Methods:** We administered a single dose of i35-Bregs (1.5×10^6^) to mice undergoing allo-HSCT and monitored disease severity and survival of GVHD mice over 90 days post-transplantation. We discovered that i35-Bregs secrete exosomes containing membrane-bound IL-35 (i35-Exosomes) and investigated whether *ex-vivo* generated i35-exosomes can be used as stand-alone immunotherapy for GVHD. i35-Breg-induced expression of cytokines or checkpoint proteins (PD-1, LAG-3, CTLA-4) was analyzed by Flow cytometry, ELISA, and RNA-seq analysis. Characterization of membrane-bound IL-35 was by Proximity ligation assay (PLA), immunohistochemistry/Confocal microscopy and Alpha Fold-Multimer modeling.

**Results:** A single dose of 1.5×10^6^ i35-Breg reduced severity of GVHD and prolonged GVHD survival, with more than 70% i35-Breg-treated mice surviving beyond day-90 post-transplantation while observing 100% mortality among untreated mice by day-45. Contrary to the view that IL-35 is secreted cytokine, we show here that i35-Bregs mitigate GVHD via membrane-bound IL-35 and by secreting i35-exosomes. Furthermore, i35-Bregs or *ex-vivo* generated i35-exosomes induce alloreactive T-cells to upregulate checkpoint proteins associated with T-cell exhaustion and anergy, inhibiting alloreactive responses and propagating infectious-tolerance mechanisms that suppress GVHD. Importantly, i35-Bregs or i35-exosomes suppresses GVHD by increasing bystander lymphocytes coated with immunosuppressive i35-exosomes.

**Conclusions:** This study demonstrates that i35-Bregs and i35-exosomes play a critical role in mitigating GVHD. The combination of i35-Breg and i35-exosome immunotherapy may be an effective strategy for treating GVHD and other inflammatory diseases.

## Introduction

Allogeneic hematopoietic stem cell transplantation (allo-HSCT) is a therapeutic option for many hematological malignancies, but its efficacy is limited by graft-versus-host disease (GVHD). GVHD can affect mucosal surfaces, skin, lungs, liver, gut, eyes and is classified into two forms based on the duration of the disease, severity of the symptoms and the tissues affected 1, 2. Acute GVHD (aGVHD) is caused primarily by alloreactive T cell responses to host tissues while chronic GVHD (cGVHD) affects 50-80% of long-term survivors of allo-HSCT and has a high mortality rate. In contrast to aGVHD caused by direct T cell recognition of histocompatibility antigens by alloreactive lymphocytes, cGVHD resembles classical T-cell/antibody-mediated autoimmune diseases like systemic lupus erythematosus, rheumatoid arthritis and Sjogren's syndrome that are mediated by both alloreactive T cells and B cells 3-8, with the involevement of B cells in antigen presentation to T cells, germinal center formation and autoantibody production, as demonstrated in murine models 9, 10. Human studies further support these findings, highlighting the contributions of B cells in cGVHD pathogenesis 11-13. Steroids are the primary and first-line treatment for GVHD although second-line treatments include, calcineurin inhibitors, mTOR-inhibitors, and cytostatic agents. Although, steroids are most effective, steroid-refractory GVHD is not infrequent and is associated with very high mortality rates 14, 15. Taken together, steroids and immunosuppressive therapies that target T cells have significant adverse effects and are the impetus to develop safer therapies for GVHD.

Interleukin 10 (IL-10)-producing regulatory T cells (Treg) or B cells (Breg) have been reported to play critical roles in immune modulation in mouse and human studies 8. Similarly, IL-35-producing regulatory B cells (i35-Bregs) or IL-35-producing Tregs (iT_R_35) suppress autoreactive lymphocytes that mediate autoimmune diseases 6, 7 and reduced levels of IL-35 in GVHD patients correlate with GVHD pathogenesis, underscoring therapeutic potential of i35-Bregs or iT_R_35 immunotherapy in GVHD 16, 17. Despite critical roles of IL-35 in suppressing inflammatory diseases 18-20 and promoting tumor metastasis by suppressing cytotoxic CD8^+^ anti-tumor T cells, gaps in mechanistic understanding of how IL-35 mediates immunosuppression have prevented its therapeutic use in humans. IL-35 is a heterodimeric cytokine comprised of two subunits, IL-12p35 (p35) and Epstein-Barr virus induced gene 3 (Ebi3). Because *Il12a* and *Ebi3* genes encode distinct signal peptides, it has been assumed that each subunit is secreted as an independent protein which eventually associate *in vivo* to produce the functional heterodimeric IL-35. Surprisingly, recent reports have suggested that p35 and Ebi3 are not necessarily secreted as the heterodimeric IL-35 cytokine but it is instead proposed that p35 and Ebi3 are independent anti-inflammatory cytokines 21-23.

In this report, we have used IL-35-producing B cells that regulate immunity during autoimmune diseases to investigate whether i35-Breg immunotherapy can be used to treat aGVHD. We also performed detailed biochemical and functional analyses of the i35-Breg cell to address the question whether i35-Bregs suppress inflammation by secreting p35 and Ebi3 as independent cytokines 22, or by secreting the heterodimeric IL-35. We show that i35-Bregs prolong survival of aGVHD mice, suppress and ameliorate GVHD via membrane-bound IL-35 and by secreting exosomes with membrane-bound IL-35. We also show that either i35-Bregs or *ex-vivo* generated i35-exosomes suppress aGVHD by propagating infectious tolerance mechanisms that increase *in vivo* levels of bystander suppressor lymphocytes coated with immunosuppressive i35-exosomes.

## Results

### IL-35-producing B cells (i35-Bregs) alleviate and prolong survival of GVHD mice

**Figure 1:**
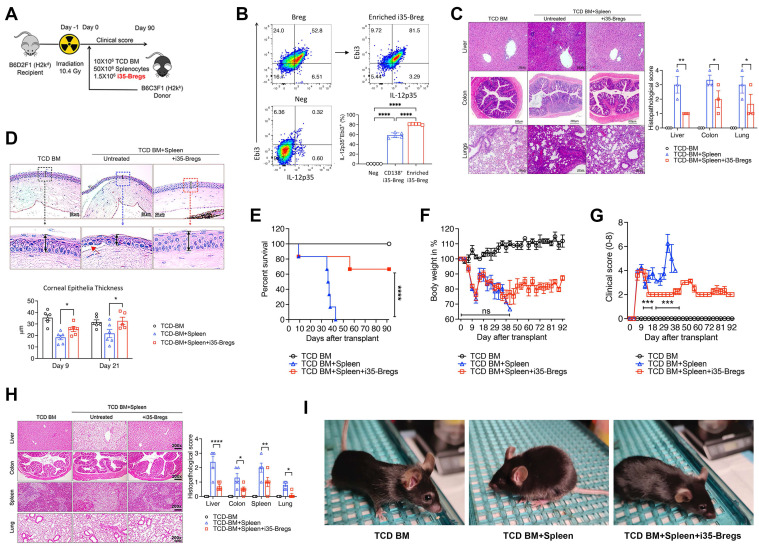
** i35-Breg cells suppress GVHD-related morbidity and mortality in mice. (A)** 10-12-week-old recipient female B6D2F1/Crl (H2k^d+^H2k^b+^, *n* = 6/group) mice were irradiated (10.4 Gy) and received 10×10^6^ T cell-depleted bone marrow cells (TCD-BM) or TCD-BM plus 50×10^6^ spleen cells (TCD-BM+SP) from donor B6C3F1/Crl (H2k^k+^H2k^b+^) mice. The treatment group received 10×10^6^ TCD-BM+SP and 1.5×10^6^ i35-Bregs (TCD-BM+SP+i35-Breg) from donor mice. **(B)** B cells from donor mouse spleen were enriched for IL-35-expressing B cells (i35-Bregs) and only preparations containing more than 80% i35-Bregs were used in these studies. **(C-D)** Tissues were harvested 21 days after allogeneic transplantation and paraffin-embedded sections were stained with Hematoxylin and eosin (H&E), magnification ×200 and scale bar 200 µm). Masked pathologists examined the H&E-stained sections and provided the histopathology scores for the liver, colon and lung, n=3 /group **(C)**. Corneal epithelia thickness was measured on Zeiss software **(D)**. To determine long-term efficacy of i35-Breg therapy, GVHD disease progression was assessed up to day-90 post-transplantation. Mortality **(E)**, body weight **(F)** and disease score **(G)** were monitored and assessed by masked investigators. **(H)** Tissues were harvested 9 days after allogeneic transplantation and paraffin-embedded sections were stained with Hematoxylin and eosin (H&E), magnification ×200 and scale bar 200 µm). Masked pathologists examined the H&E-stained sections and provided the histopathology scores. **(I)** Representative phenotypes of the TCD-BM, TCD-BM+Spleen and TCD-BM+Spleen+i35-Breg treated mice. Data represent mean ± SEM and 3 independent experiments. *P < 0.05; **P < 0.01; ***P < 0.001; ****P < 0.0001.

We used an MHC-haploidentical murine allogeneic bone marrow transplantation model to assess whether i35-Breg would be effective in suppressing aGVHD 24. The haploidentical B6C3F1 > B6D2F1 aGVHD model was selected for this study because it reproduces key immunopathological features of GVHD, including chronic multi-organ inflammation and immune dysregulation involving both T cells and B cells. Recipient mice received 10.4 Gy total body irradiation and within 12 h the recipient B6D2F1/Crl (H2k^d+^H2k^b+^) mice were transplanted with T-cell depleted (TCD) bone marrow cells (TCD BM) or TCD BM plus spleen cells (TCD BM+spleen) from B6C3F1/Crl (H2k^k+^H2k^b+^) donor mice (Figure 1A). Mice in the treatment group received TCD BM+spleen with 1.5×10^6^ i35-Breg cells (TCD BM+spleen+i35-Bregs). The i35-Bregs were *ex-vivo* generated from activated B cells, purified by magnetic-bead sorting and characterized by intracellular cytokine staining assay. More than 81.5% of the B cells were IL-35 producing B cells (Figure 1B) and each i35-Breg preparation used for treatment was tested for immunosuppressive activity by *in vitro* T cell proliferation inhibition assay as described 25, 26. CD19^+^ B cells that do not express IL-35 were used as controls and these cells do not suppress T cell proliferation *in-vitro* or *in-vivo* 25, 26. Histopathological analysis of the lungs, liver, colon and eyes harvested on day-21 post-transplantation was performed (HistoServe Inc., Germantown MD) and the histopathology scores show significantly reduced pathology in tissues of mice treated with i35-Breg cells (Figure 1C). GVHD-associated dry eye disease is the most common ocular condition that develops after allogeneic transplantation and is characterized by extensive destruction of ocular epithelial tissues including the cornea, conjunctiva, and lacrimal gland. In contrast to the significant thinning of the corneal epithelium and epithelial erosion (red arrow) observed in the untreated mice with GVHD, the i35-Breg-treated mice do not exhibit these pathognomonic features of GVHD-associated dry eye disease, underscoring the protective effect of i35-Breg immunotherapy (Figure 1D). We next investigated the long-term efficacy of i35-Breg therapy by assessing effects of i35-Breg on GVHD disease progression, clinical symptoms and pathological changes in the liver, colon, spleen and lung 3 months after allogeneic transplantation. Mice that received TCD BM+Spleen developed severe GVHD and died within 45 days of receiving the allograft (Figure 1E). In contrast, treatment with i35-Breg cells prolonged life of the GVHD mice, with more than 70% of the mice surviving beyond 90 days post-transplantation (Figure 1E). The mice were scored on five clinical parameters (body weight change, posture, activity, fur texture, and skin integrity) according to the Cooke *et al.* scoring system, and the GVHD clinical scores were generated by summation of the five parameters as described 27.

The i35-Breg-treated mice maintained 80% of their body weight with relatively mild clinical symptoms (Figure 1F-G). The histopathology scores of mice treated with i35-Breg cells show significantly reduced pathology (Figure 1H). Because only 1-2% of all B cells in the spleen are regulatory lymphocytes 28, 29, the suppression of GVHD is attributable to the 1.5×10^6^ i35-Bregs transferred to mice in the treatment group. Photographic images also show that the control (TCD BM) and i35-Breg treated GVHD mice exhibit normal phenotype while the untreated GVHD mice (TCD BM+Spleen) exhibit a hunched posture, ruffled fur, squinted eyes and reduced movement, which are symptoms of significant morbidity among GVHD mice (Figure 1I). Taken together, these observations provide evidence for efficacy of i35-Breg immunotherapy, at least in mouse GVHD.

### i35-Bregs inhibit Th1 and Th17 cells and induce expansion of Treg and Breg cells

**Figure 2:**
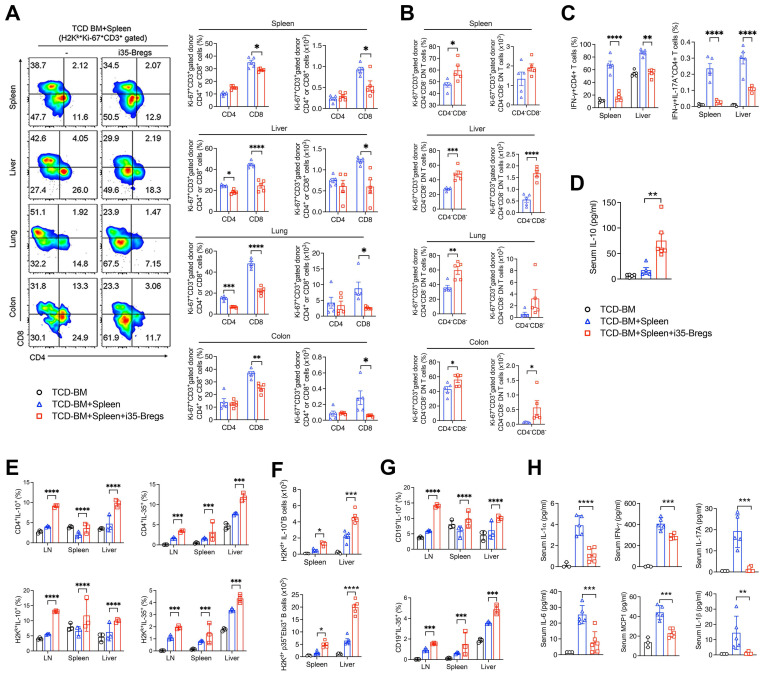
** i35-Breg cells inhibit alloreactive T cells and induce Treg and Breg expansion.** Lymphocytes isolated from tissues were analyzed by FACS. **(A-B)** Representative flow plots show proliferation of donor (H2k^k+^) CD3^+^CD4^+^, CD3^+^CD8^+^ or CD3^+^CD4^-^CD8^-^ DN T cells (*n*=>4/group). **(B)** Bar graphs show percentage of H2k^k+^Ki-67^+^CD3^+^CD4^+^, H2k^k+^Ki-67^+^CD3^+^CD8^+^ or H2k^k+^Ki-67^+^CD3^+^CD4^-^CD8^-^ DN T cells. **(C)** Lymphocytes were analyzed by intracellular cytokine staining assay and bar charts show percent CD4^+^ T cells expressing IL-17 and/or IFN-γ in the spleen or liver **(C, Figure S1A)**; percent Ki-67^+^Foxp3^+^, Foxp3^+^CD25^+^, or IL-10-expressing Foxp3^+^ CD4^+^ T cells **(Figure S3A)**. **(D)** Quantification of IL-10 in the serum of mice on day-9 post-transplantation by ELISA (*n*=5/group). **(E)** Lymphocytes isolated from tissues on day-21 post-transplantation were analyzed by FACS (*n*=3/group). Bar charts show representative results of percent donor (H2H^k+^) CD4^+^ T cells expressing IL-10 or IL-35 in the lymph nodes (LN), spleen and liver. **(F-G)** Bar charts show absolute numbers or percentages of recipient (H2H^d+^) B lymphocytes expressing IL-10 or IL-35 in the LN, spleen or liver on day-9 **(F)** or day-21 post-transplantation **(G, Figure S3B)***.* **(H)** Sera were obtained from the mice (*n*=5/group) 9 days after allogeneic cell transfer and serum level of the indicated cytokines was determined by multiplex ELISA. Data represent mean ± SEM and three independent experiments. *P < 0.05; **P < 0.01; ***P < 0.001; ****P < 0.0001. For the gating strategy of CD3^+^ cells, see **Figure S1A-B**).

Radiation-induced tissue damage activates a variety of immune cells that secrete large amounts of inflammatory cytokines associated with cytokine storm and GVHD pathology 2. We gated proliferating lymphocytes (Ki67^+^) in the liver, lung, colon and spleen (Figure S1A) and FACS analyses of CD3^+^ T-lymphocytes in these tissues on day-9 after allogeneic transplantation, reveal significant reduction of CD4^+^ and CD8^+^ T cells in the mice treated with i35-Breg cells (Figure 2A), indicating a correlation between i35-Breg treatment and inhibition of alloreactive lymphocytes. Surprisingly, we observed significant increase of CD3^+^CD4^-^CD8^-^ T cells (DNTs) in the liver, lung and colon of mice that received i35-Breg therapy (Figure 2B). It is of note that allogeneic DNTs evade rejection by host immune cells 30 while infusion of allogeneic DNTs induces long-term survival of cardiac, skin, and islet grafts 12, 31, 32. Importantly, the DNT subpopulation has also been shown to suppress GVHD in mice and humans 33, 34. Suppression of GVHD in mice treated with i35-Bregs also correlates with decrease of proinflammatory lymphocyte subsets that produce IFN-γ (Th1) or both IL-17A and IFN-γ (Th17-DP) in the liver and spleen (Figure 2C and Figure S2A). Furthermore, reduced levels of Th1 and Th17 cells is consistent with lower percentages of cells expressing the Th17 or Th1 lineage-determining transcription factors, ROR-γT or T-bet (Figure S2B*).* The decrease of Th17-DP cells is notable because elevated level of Th17-DP is associated with severe inflammatory diseases including, multiple sclerosis 35, Crohn's disease 36, and uveitis 22. On the other hand, amelioration of GVHD correlates with the secretion of IL-10 in the serum of mice that received i35-Breg immunotherapy (Figure 2D). We also observed expansion of IL-10- and IL-35-expressing Tregs in the liver, lymph nodes, spleen or lung of mice treated with i35-Bregs (Figure 2E and Figure S3A), underscoring the roles of these immunosuppressive cytokines in mitigating GVHD.

While the IL-10-producing T cells (Treg/Tr1) are the best characterized regulatory lymphocyte population 37, regulatory B cells (Bregs) that suppress inflammation and autoimmune diseases through production of IL-10 (i10-Bregs) or IL-35 (i35-Bregs) have also been described 25, 38-40. We observed significant expansion of i10-Breg and i35-Breg cells in the liver, spleen or lymph nodes on day-9 (Figure 2F and Figure S3B) and day-21 (Figure 2G) of mice treated with i35-Breg cells but not the untreated mice. Although the i35-Bregs transferred during treatment were of the H2k^k+^ haplotype, we observed significant expansion of IL-10- and IL-35-producing Bregs of the H2k^d+^ haplotype in the liver, LN and spleen of the recipient mice (Figure 2G). This observation suggests that the 1.5×10^6^ i35-Breg cells transferred to the recipient mice were sufficient to promote infectious tolerance, a natural *in vivo* process whereby a few number of regulatory lymphocytes can convert bystander lymphocytes to acquire capacity of producing immune suppressive cytokines that promote a tolerance-inducing environment 41-45. Consistent with the expansion of immunosuppressive lymphocytes, serum levels of IL-6, IL-17A, IFN-γ, MCP-1, IL-1α and IL-1β are significantly reduced in i35-Breg-treated mice (Figure 2H) indicating that i35-Breg cells might suppress the production of cytokine storms implicated in GVHD pathology.

### i35-Bregs that suppress GVHD express PD-1, LAG-3 and CTLA-4 checkpoint proteins

**Figure 3:**
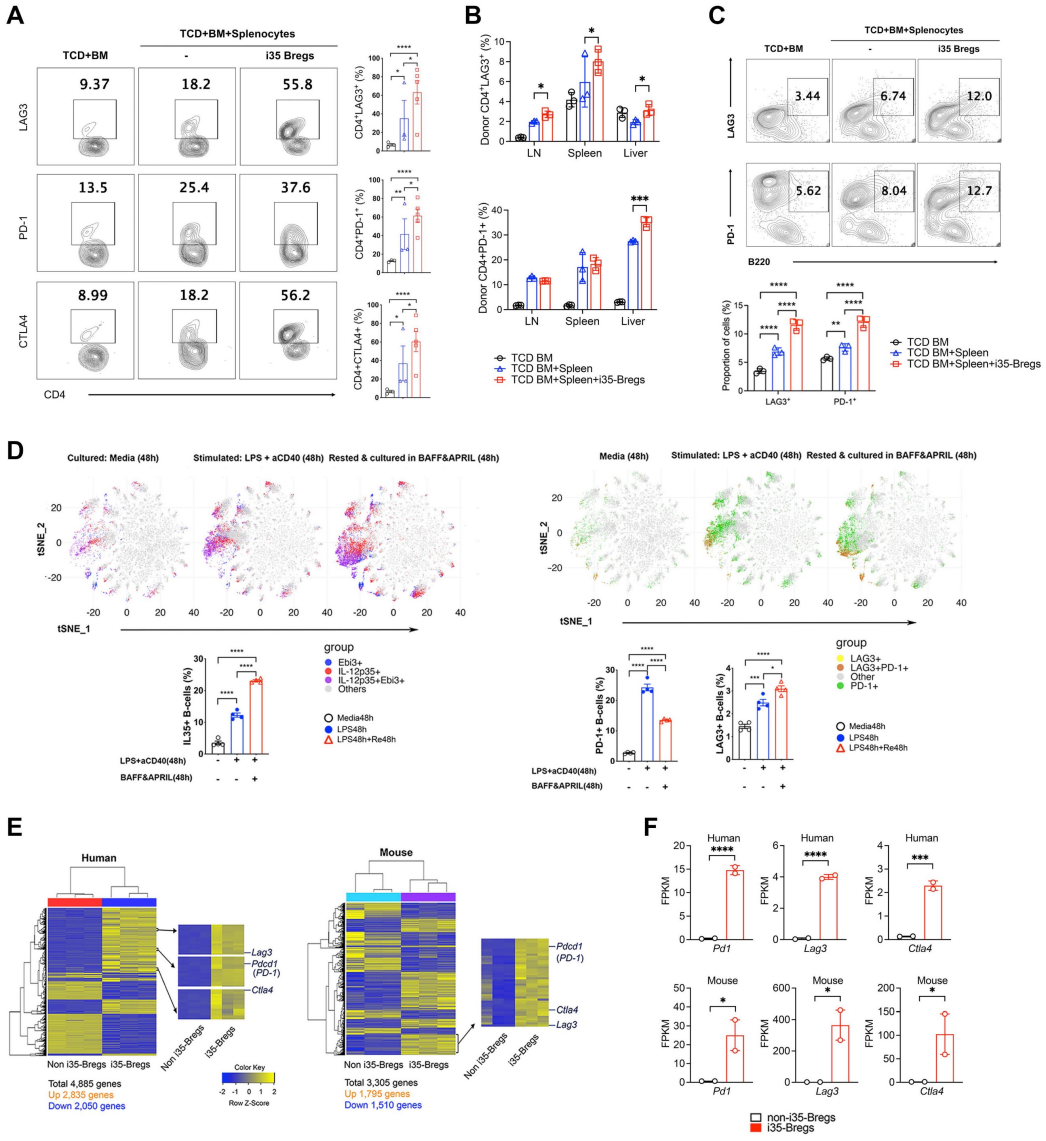
** i35-Bregs constitutively express PD-1 and LAG-3. (A-B)** CD4^+^ T-cells isolated on day-9 **(A)** or day-21 **(B)** post transplantation from GVHD mice untreated or treated with i35-Bregs and subjected to FACS analysis. Numbers in quadrants indicate percent CD4^+^ T-cells in spleen, LN or liver expressing cell-surface LAG-3, PD-1 or CTLA-4 (*n* = 3-5). **(C)** B220 cells isolated on day-30 post transplantation from GVHD mice were untreated or treated with i35-Bregs and subjected to FACS analysis. Numbers in quadrants indicate percent B220^+^ cells in spleen and LN expressing cell-surface LAG-3 or PD-1 (*n* = 3). **(D)** CD19^+^ B-cells from spleen were stimulated with LPS/anti-CD40 and maintained for 96h in medium containing BAFF/APRIL. Representative t-SNE clustering plots show spatial localization and abundance of B-cells expressing IL-35 (D, left panel) or PD-1 and/or LAG3 (D, right-panel). **(E-F)** IL-35^-^CD19^+^ B-cells (non-i35-Bregs) (*n* = 3) and IL-35^+^-CD19^+^ B-cells (>83% i35-Bregs) (*n* = 3) were isolated from human PBMC or mouse spleen, sorted and characterized as described 55. RNA from cells were subjected to RNA-seq analysis and i35-Breg and non-i35-Breg transcriptomes were compared. **(E)** Differentially expressed genes (DEG) heatmaps show that *Pd1*, *Lag3* and *Ctla4* are preferentially expressed by i35-Breg but not non-Breg cells. **(F)** Representative graphs from RNA-seq analysis show FPKM expression values for *Ctla4*,* Lag3*, and *Pd1* expressed genes. Y-axis represents FPKM expression level, and X-axis represents the gene name. Graph color represents non-i35-Bregs (black) or i35-Bregs (red). Data represent mean ± SEM. *P < 0.05; **P < 0.01; ***P < 0.001; ****P < 0.0001.

Checkpoint proteins including programmed cell death protein 1 (PD-1), lymphocyte activation gene 3 protein (LAG-3), cytotoxic T‑lymphocyte-associated antigen 4 (CTLA-4) play important roles in peripheral tolerance and elevated cell-surface expression of PD-1 or LAG-3 is a hallmark feature of exhausted CD4^+^ and CD8^+^ T cells, impaired T cell proliferation and diminished effector functions 46-48. In this study, we observed significant increase of LAG-3^+^, PD-1^+^ or CTLA-4^+^ T cells in the liver, spleen and lymph nodes of i35-Breg-treated mice on day-9 (Figure 3A) and day-21 (Figure 3B*)* post transplantation. This observation correlates with reduced levels of proinflammatory cytokines in the serum of mice that received i35-Breg immunotherapy (Figure 2H), suggesting that i35-Breg cells might suppress GVHD by inducing T cell exhaustion and rendering allogeneic T cells less functionally active. Interestingly, while expression of LAG-3 or PD-1 is a marker of exhausted T cells 20, increased cell-surface expression of LAG-3 and/or PD-1 is a marker of regulatory B cell subpopulations with enhanced suppressive activities and therapeutic efficacy 49-53. Also, IL-10^+^ Tregs with intrinsic capacity to suppress inflammation are distinguishable from otherwise proinflammatory cells by co-expresssion of these co-inhibitory receptors 54. Consistent with tolerogenic and immune-suppressive effects of checkpoint proteins, we detected significant increase of PD-1^+^ and LAG-3^+^ B cells in GVHD mice treated with i35-Bregs (Figure 3C). Moreover, analysis of *in-vitro* activated CD19^+^ B cells revealed progressive increase of IL-35^+^, PD-1^+^ and LAG3^+^ B cells at various time points during Breg development and differentiation. By day-4, more than 25% of the cells in culture were IL-35^+^ B cells (Figure 3D) with percentage of IL-35^+^ B cells exceeding 85% after 2-3 cycles of activation and resting in medium containing Baff and April as previously reported 25, 55. Importantly, the IL-35^+^ B cells (i35-Bregs) co-localize spatially with PD-1^+^ or LAG-3^+^ B cells in tSNE plots (Figure 3D), suggesting that i35-Breg cells might acquire capacity to express PD-1 and/or LAG-3 during their differentiation or development. Likewise, IL-35-producing human B cells also express PD-1 checkpoint protein (Figure S4). We confirm that i35-Bregs constitutively express checkpoint proteins by transcriptome analyses using RNA from IL-35-negative B cells and i35-Breg cells (>83% i35-Bregs) as described 26, 55. Results of the RNA-seq analyses also confirm that both human and mouse i35-Bregs constitutively express *Pd1, Lag3 and Ctla4* (Figure 3E-F).

### i35-Breg cells express membrane-bound IL-35

**Figure 4:**
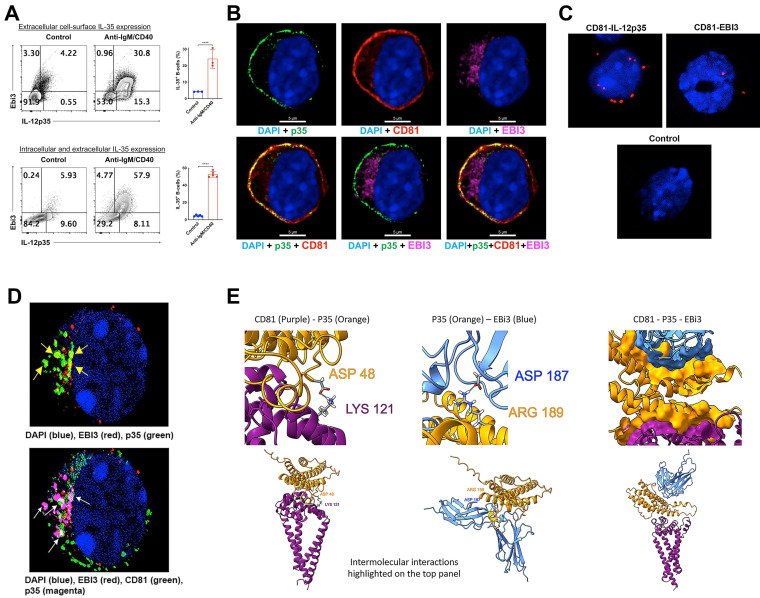
** i35-Breg cells express membrane-bound IL-35.** Mouse and human CD19^+^ B cells were activated with anti-IgM or CpG respectively for 72 h in medium containing anti-CD40 Abs. **(A)** For detection of membrane-bound IL-35 (p35^+^ and Ebi3^+^), the cells were analyzed by FACS without permeabilization. For detection of intracellular IL-35 expression, the cells were permeabilized and then subjected to intracellular cytokine staining assay. **(B)** Activated CD19^+^ B cells were analyzed for cell surface expression of p35, Ebi3 or CD81 by immunohistochemical staining and confocal microscopy. **(C)** Proximity Ligation Assay (PLA) was used to demonstrate physical interaction between CD81 and p35 or Ebi3 on the plasma membrane. Sections were counter-stained with DAPI and the red dots detection of CD81 within <40 nm of p35 on the B cell membrane. **(D)** Confocal images showing polarized clustering of p35 and Ebi3 (Top panel) or p35, Ebi3 and CD81 (Bottom panel) on the plasma membrane of B cells stimulated with anti-IgM and anti-CD40 Abs for 72 h. **(E)** Interactions between CD81, p35 and Ebi3 was modeled using AlphaFold2-multimer with relaxation. (Left panel) interactions between p35 and CD81; (Middle panel) interactions between p35 and Ebi3; (Right panel) interactions between p35, Ebi3 and CD81. Data represent at least three independent experiments. Data represent mean ± SEM and three independent experiments. ***P < 0.001; ****P < 0.0001.

The prevailing opinion that IL-35 is a secreted cytokine is based on *in vitro* studies which showed that p35 and Ebi3 subunits have distinct signal peptides that promote their secretion. It was therefore assumed that p35 and Ebi3 eventually associate *in vivo* by unknown mechanisms to produce the functional heterodimeric IL-35 cytokine that suppress inflammation. However, recent reports indicate that p35 and Ebi3 are not necessarily secreted as a heterodimer but as independent cytokines with intrinsic immunosuppressive activities 21, 22. We therefore investigated whether suppression of GVHD by i35-Bregs is mediated through secretion of p35 and/or Ebi3 cytokines or by secretion of the heterodimeric IL-35 cytokine. The i35-Breg cells used in these studies were produced *ex-vivo* by activating naive mouse CD19^+^ B cells with anti-IgM/anti-CD40 for 72 h. The cells were then permeabilized, subjected to intracellular cytokine staining assay and FACS analysis revealed that the total percentage of p35^+^Ebi3^+^ Bregs (intracellular + membrane-bound IL-35) was ~57.9% (Figure 4A; bottom panel). To quantify the percentage of B cells expressing membrane-bound IL-35, we stained the cells with p35 and Ebi3 antibodies without permeabilizing, followed by direct cell surface FACS analysis. We found that ~30.8% of the B cells expressed both p35 and Ebi3 on their cell-surface (Figure 4A; top panel) and these observations were confirmed using human i35-Bregs generated by stimulating human CD19^+^ B cells with CpG for 72 h. Similar to mouse i35-Breg cells, ~40% of the human i35-Bregs expressed membrane-bound p35 and Ebi3 (Figure S5). We also confirm that i35-Breg cells indeed express cell-surface IL-35 by immunohistochemical and confocal microscopy analyses. Surprisingly, expression of the p35 subunit localized to the plasma membrane in association with CD81, a tetraspanin trans-membrane protein and component of the B cell receptor (BCR) co-receptor complex (Figure 4B) 56, 57. In contrast to the membrane-bound p35, the Ebi3 protein was barely detectable on the plasma membrane but localized mainly to the lumen of the endoplasmic reticulum (ER). This pattern of Ebi3 expression is characteristic of secreted proteins and conforms to the co-translational model whereby the signal peptide is removed concomitant with translocation into the ER and eventual secretion 58-60. Proximity ligation assay (PLA) is a more powerful *in-situ* protein-protein interaction detection method compared to FRET (Förster resonance energy transfer). It uses single-stranded oligonucleotides conjugated to target proteins to identify physical closeness of proteins within 40 nm in a tissue section or membrane. We used PLA to demonstrate physical interaction between p35 and CD81 on the cell surface of i35-Breg cells while interaction between Ebi3 and CD81 was barely detectable (Figure 4C), providing direct evidence that p35 is expressed as a transmembrane protein while Ebi3 is a secreted protein. However, prolonged stimulation of i35-Breg cells with BCR agonists induces polarized localization of Ebi3 to the plasma membrane where it interacts with p35:CD81 to form BCR microdomains containing a p35:Ebi3:CD81 complex (Figure 4D). We also used the Alpha Fold-Multimer modeling program to predict intermolecular interactions between p35, CD81 and Ebi3 proteins 61. The CD81:p35 dimer model predicts formation of an isopeptide covalent bond between CD81:Lys-121 and IL-12p35:Asp-48 that conceivably mediates the stable interaction between p35 and CD81 on the i35-Breg plasma membrane (Figure 4E*;* Left panel). Besides the isopeptide bond, the model also predicted additional interface contact residues (not shown). While the p35:Ebi3 dimer model predicts intermolecular interaction between ASP-187 of p35 and Ebi3 ARG-189, the score for interaction between CD81 and Ebi3 is lower consistent with results of our PLA data. Nonetheless, the CD81:p35:Ebi3 trimer model predicts that while Ebi3 interacts with p35, it does not interact directly with CD81 (Figure 4E, Right panel). None of the relaxed models had any violations.

### Exosomes with membrane-bound IL-35 (i35-Exosomes) suppress GVHD

**Figure 5:**
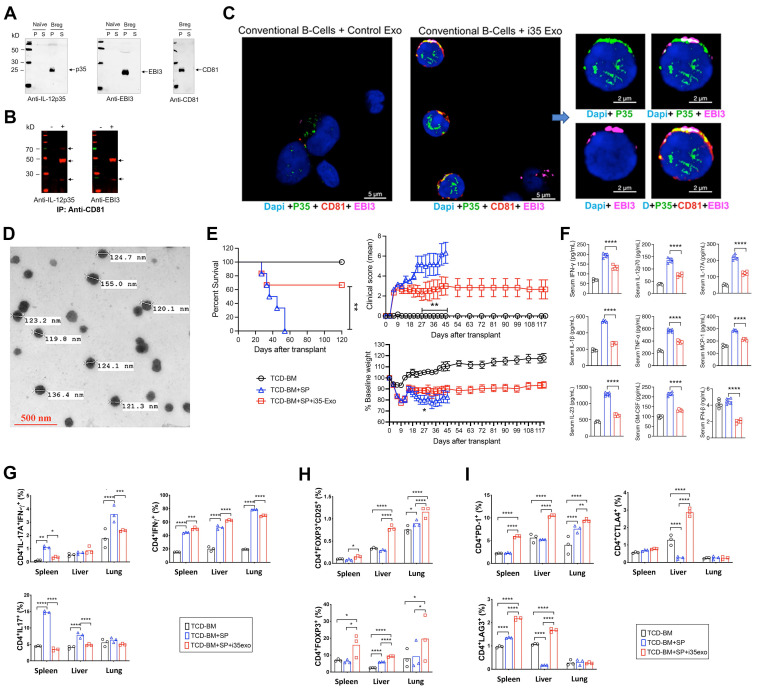
** Exosomes with membrane-bound IL-35 (i35-Exosomes) suppress GVHD. (A-B)** CD19^+^ B-cells were activated for 72h in medium containing anti-IgM/anti-CD40 Abs. **(A)** Supernatants from the cultured cells were subjected to ultracentrifugation (100,000xg) and soluble (S) or particulate (P) fractions analyzed by Western blotting. (**B**) p35 or Ebi3 was immunoprecipitated with Anti-CD81 and precipitated proteins were analyzed by Western blotting. **(C)** Conventional B-cells were cultured with i35-exosomes or Control exosomes for 72 h. Co-localization of p35 and Ebi3 on cell-surface of the conventional B-cells was detected by immunohistochemical/confocal microscopy analyses.** (D-I)** GVHD was induced in irradiated B6D2F1/Cr1 (H2K^d+^) mice by transplanting 10×10^6^ TCD-BM cells or TCD-BM+50×10^6^ spleen cells (TCD-BM+SP) from donor B6C3F1/Cr1 (H2K^k+^) mice. The treatment group were treated similarly with TCD-BM+SP but also received 2×10^10^ i35-exosomes (20 ng) (TCD-BM+SP+i35-exo) by intravenous injection on day 0, 3, 6, 9, 12 post-transplantation. **(D)** Photomicrograph shows sizes of i35-exosomes used for treatment, as determined by transmission electron microscopy (40,000x magnification) (see Figure S7*)*. **(E)** GVHD mortality, body weight and disease score were monitored and assessed by masked investigators. For GVHD experiments, we used n=7-10 per group. Survival, clinical scores and body weight were monitored for 120 days, post-transplantation. **(F)** Serum-cytokine levels at day-30 post-transplantation were quantified by multiplex ELISA. **(G-I)** Lymphocytes from spleen, liver, or lung were analyzed by intracellular cytokine assay and/or cell-surface FACS. Bar charts show percent CD4^+^ T-cells expressing IL-17A and/or IFN-γ **(G)**, Foxp3 and/or CD25 **(H)**, PD-1, LAG3 or CTLA-4 **(I)**. Data represent mean ± SEM and three independent experiments. *P < 0.05; **P < 0.01; ***P < 0.001; ****P < 0.0001.

In addition to expressing membrane-bound IL-35, i35-Breg cells also secrete IL-35-containing exosomes (i35-exosomes) 62. We therefore investigated whether i35-exosomes can be used as a stand-alone immunotherapy to suppress GVHD. Supernatants from cultured i35-Breg cells were harvested, cell debris were removed by low-speed centrifugation, followed by ultracentrifugation at 100,000 xg. Western blot analysis shows that p35, Ebi3 and CD81 preferentially localized to the particulate but not the soluble fraction (Figure 5A). The particulate fraction was resuspended in PBS and subjected to immunoprecipitation analysis. Antibodies specific to CD81 immunoprecipitated p35 or Ebi3 indicating that i35-exosomes contain membrane-bound IL-35 in association with CD81 (Figure 5B). Interestingly, when we co-cultured the i35-exosomes with conventional CD19^+^ B cells for 72 h and stained the cells with fluorescent-labeled antibodies, p35, Ebi3 and CD81 co-localized to the B cell plasma membrane (Figure 5C and Figure S6), suggesting uptake of the i35-exosomes by the B cells. This observation is akin to a recent study showing that bystander T cells coated with IL-35^+^ extracellular visicles (EVs) secreted by Tregs propagate infectious tolerance mechanisms that suppress inflammation 44, 63, 64. We therefore investigated whether the i35-Breg cells also suppress GVHD by secreting and coating (cross-dressing) bystander conventional lymphocytes with i35-exosomes. To directly examine whether i35-exosomes can be used as stand-alone immunotherapy for GVHD, we isolated i35-exosomes from highly enriched i35-Breg cells preparations; tested for capacity to express IL-35 and suppress lymphocyte proliferation *in-vitro* (T cell proliferation assay) according to our standard operating procedure 25. Detailed description of EV separation, characterization, quantification and determination of i35-exosome size distribution is provided (Materials and methods, Figure S7). The i35-exosomes used for treatment ranged in size from 119.8 nm to 155 nm as determined by electron microscopy (Figure 5D) and the amount of IL-35 contained in the exosomes was established by ELISA. Each mouse in the treatment group received intravenous injection of 2×10^10^ i35-exosomes (20 ng, IL-35) on days 0, 3, 6, 9, 12 post-transplantation and GVHD disease was scored for body weight changes, posture, activity, fur texture, and skin integrity as described 27, 65. By day-55 post-transplantation, we observed 100% mortality among untreated mice while >60% of i35-exosome treated mice survived beyond day-120, with clinical scores indicating attenuation of GVHD (Figure 5E). Sera collected on day-30 post-transplantation show significant elevation of IFN-γ, IL-12p70, IL-17A, IFN-β, IL-1β, TNF-α, MCP-1, IL-23, GM-CSF and TNF-α in the untreated mice while levels of these cytokines are significantly reduced in mice that received i35-exosome (Figure 5F). Intracellular cytokine analyses show significant reduction in the percentages of IL-17A^+^ and IL-17A^+^IFN-γ^+^CD4^+^ T cells in spleen, liver and lung of i35-exosome-treated mice (Figure 5G and Figure S8A) which coincides with expansion of Treg cells (Figure 5H and Figure S8B). Similar to i35-Breg treatment, we observed significant increase of PD-1^+^, LAG-3^+^ or CTLA-4^+^ T cells in mice treated with i35-exosomes (Figure 5I and Figure S8C-E), suggesting that i35-exosomes suppressed GVHD in part by inducing increase in percentage of allogeneic T cells with upregulated expression of checkpoint proteins. While the immunosuppressive effects of i35-Bregs and i35-exosomes provide strong therapeutic potential for mitigating GVHD, the potential effects of i35-Bregs or i35-exosomes on the graft-versus-leukemia (GVL) activity is of major concern when evaluating the safety profile of immunosuppressive agents employed to treat GVHD. Although the present study focused on the mechanisms by which i35-Bregs and i35-exosomes mitigate GVHD, future investigations will examine whether they interfere with GVL effect. Such studies are essential in establishing i35-Breg-based immunotherapy as a safe and effective alternative to current immunosuppressive strategies.

## Discussion

Allogeneic hematopoietic cell transplantation (allo-HSCT) can be a lifesaving intervention for patients with aggressive hematologic malignancies. However, a serious and challenging complication of allo-HSCT is the risk of developing GVHD, a debilitating disease with limited therapeutic options 14, 15. Steroids are most effective, but their adverse effects have been the impetus to develop safer alternatives, including cellular immunotherapies. In preclinical and clinical studies, *ex-vivo* generated IL-10-producing regulatory lymphocytes have been shown to ameliorate GVHD in mice and human but major obstacles to clinical use of Tregs include the high dose of Treg cells required and difficulty of producing large amounts of Tregs *ex-vivo* 66, 67.

In this study, we show that administering a single dose of 1.5×10^6^ i35-Breg is sufficient to prolong the survival of GVHD mice beyond day-90 of allograft transplantation while 100% mortality rate is observed among the untreated mice by day 45. We show that the i35-Bregs suppress alloreactive responses by inducing alloreactive T cells to upregulate checkpoint proteins (PD-1, LAG-3) associated with T-cell exhaustion/anergy, suggesting that efficacy of i35-Breg immunotherapy may derive in part from reduced capacity of the alloreactive T cells to induce cytotoxicity or produce cytokine storms that exacerbate GVHD. While LAG-3^+^/PD-1^+^ T cells are associated with T cell exhaustion and anergy, LAG-3^+^/PD-1^+^ B cells have been identified as specialized regulatory B cell subpopulations with enhanced immunosuppressive activities 49, 51, 52. Moreover, co-expression of immune checkpoint proteins has been identified as a key distinguishing feature of IL-10^+^ Tregs with suppress function 54. Compared to conventional B cells, our RNA-seq and tSNE data reveal that i35-Breg cells constitutively express checkpoint proteins and finding that LAG-3^+^ and PD-1^+^ B cells are expanded in the liver and lung of mice that received i35-Breg immunotherapy, underscore the enhanced immune-suppressive activities of i35-Breg cells. Surprisingly, we also observed significant increase of CD3^+^CD4^-^CD8^-^ double negative T (DNT) cells in the liver, lung and colon of GVHD mice treated with i35-Bregs and this tissue-resident Treg subpopulation has recently been shown to suppress the severity of GVHD and other immune disorders 34, 68, 69. The correlation between the decrease of DNTs and development of severe GVHD in our study thus suggest the involvement of diverse regulatory lymphocytes in mitigation of GVHD by i35-Bregs.

i35-Breg cells also secrete IL-35-containing exosomes (i35-exosomes) and adoptive transfer of *ex-vivo* generated i35-exosomes prolongs the survival of GVHD mice, with more than 60% of i35-exosome-treated mice surviving beyond day-120 of allograft transplantation. In contrast, by day-55 post-transplantation, we observed 100% mortality rate among the untreated mice. The suppression of GVHD by i35-exosomes also correlates with increase of alloreactive T cells expressing checkpoint inhibitors (PD-1, CTLA-4, LAG-3) that promote T cell exhaustion/anergy and significant decrease of cytokines that produce cytokine storms associated with pathological responses observed in GVHD. While IL-35-containing exosomes play a critical role in the suppression of aGVHD, other mechanisms also contribute to the therapeutic effects of i35-Breg immunotherapy. These include the inhibitory effects of membrane-bound IL-35 detected on i35-Breg cells, as well as IL-35-induced upregulation of checkpoint inhibitors such as LAG3, PD-1, and CTLA4 on alloreactive T cells. These checkpoint molecules are likely involved in inducing T cell exhaustion or apoptosis, thereby reducing the pathogenic activity of alloreactive T cells. By engaging these multiple mechanisms, i35-Bregs mediate potent and multifaceted immunosuppressive effects. Interestingly, IL-35^+^ EVs secreted by Treg cells have recently been shown to coat bystander conventional lymphocytes and induce them to promote IL-35-mediated infectious tolerance mechanisms that suppress inflammatory responses 44, 45. In view of our data showing that i35-exosomes readily adhere and coat the plasma membrane of conventional B cells, it is conceivable that such “cross-dressing” of bystander lymphocytes with i35-exosomes can also propagate infectious tolerance mechanisms that contribute to suppression of GVHD by amplifying suppressive activities of IL-35. Although IL-10 and TGF-β have also been proposed as potential stand-alone treatments for GVHD, the sustained *in-vivo* production of IL-35 by i35-Bregs and i35-exosomes provides therapeutic advantage over these biologics which are rapidly cleared *in-vivo* 25, 55.

In this report, we also show that the IL-12p35 (p35) subunit is a transmembrane protein. It colocalizes on the i35-Breg plasma membrane with the tetraspanin protein CD81, which together with CD19 and CD21 comprise the B cell co-receptor complex 70. An important function of CD81 is to chaperone CD19 and co-receptor proteins through the secretory pathway, suggesting that p35 may also be chaperoned and trafficked to the B cell membrane by CD81 57, 71. We confirm that p35 indeed colocalizes with CD81 on the i35-Breg plasma membrane by immunohistochemical analysis, reciprocal immunoprecipitation analyses and proximity ligation assay. In contrast, these analyses also reveal that Ebi3 is a secreted protein and that prolonged activation of i35-Breg cells induces re-localization of Ebi3 to plasma membrane microdomains containing CD81:Ebi3:p35. AlphaFold-Multimer modelling predicts interactions between CD81 and p35 via isopeptide bonds and although Ebi3 interacts with the membrane-bound p35:CD81complex, the interaction of Ebi3 is mainly with p35 but not CD81. These observations suggest that CD81 might play essential role in the biosynthesis and function of membrane-bound IL-35 expressed by i35-Breg cells.

The CD81 tetraspanin also plays critical roles in trafficking other signaling molecules to the BCR or TCR and is suggested to function as gatekeeper of lymphocyte activation 72. In resting B cells, CD81 binds CD19, tethers it to the B cell co-receptor complex (CD19/CD81/CD21) and thereby prevents engagement of CD19 with the BCR 56, 57, 73-75. However, upon antigen stimulation, CD81 dissociates from the B cell co-receptor complex, releasing CD19 to interact with BCR and promotes B cell activation by lowering the BCR signaling threshold 75-77. Analogous to the B cell coreceptor complex, CD81 also co-localizes with TCR during T cell activation 56, 78, 79. In this study, we have shown that i35-Bregs suppress alloreactive T cells by upregulating checkpoint proteins. In contrast, we observed the expansion of Bregs in the liver, lung and spleen of i35-Breg-treated mice, implying that differential interaction of CD81 with the B or T cell coreceptor might influence signaling events that promote diametrically opposite effects downstream of TCR and BCR receptors 56, 57.

This preclinical investigation to determine whether i35-Bregs can suppress GVHD has revealed for the first time, that IL-35 is a membrane-bound cytokine, and this observation is contrary to the general belief that IL-35 and most cytokines are secreted proteins. We also show that a single dose of i35-Bregs (1.5×10^6^) is sufficient to suppress and prolong survival of aGVHD via membrane-bound IL-35. We further show that i35-exosomes secreted by i35-Breg cells can suppress GVHD by propagating infectious immunological tolerance, a physiological process whereby tolerance-inducing state is transferred from small numbers of immunoregulatory lymphocytes to conventional bystander lymphocytes 44, 45, 64. Infectious immunological tolerance was initially proposed by Gershon and Kondo in 1971 and it is now recognized as an important physiological mechanism for maintaining immune homeostasis 41, 44, 80, 81. Although it is mostly associated with Foxp3^+^ Tregs, our studies now extend that capacity of inducing infectious tolerance to i35-Bregs. However, IL-35-induced infectious tolerance is unique because it is mediated via passive transfer of i35-exosomoes to bystander conventional lymphocytes that amplify IL-35-mediated immunosuppression 44, 62, 82. Most important is our finding that treatment of aGVHD with either i35-exosome or i35-Breg does not induce observable adverse effects in mice and is effective as stand-alone therapies, implying that i35-Breg/i35-exosome combination may be a safe alternative GVHD therapy compared to steroids. Notably, other studies in mice have shown that IL-35 mitigates GVHD while preserving graft-versus-leukemia effect 19, suggesting that i35-Breg/i35-exosome immunotherapy may obviate need for long-term immunosuppression in HSCT recipients. While the current study focused on aGVHD, the immunosuppressive mechanisms mediated by i35-Bregs and i35-exosomes, particularly through membrane-bound IL-35 and infectious tolerance, may also have broader implications. Although our preclinical models were specifically designed to replicate the pathophysiology of aGVHD, our findings provide a foundation for exploring whether i35-Breg/i35-exosomes immunotherapy would be effective in cGVHD and other immune-mediated conditions. Further studies are also warranted to determine if i35-Breg/i35-exosomes immunotherapy would suppress cGVHD without compromizing GVL effects.

## Materials and methods

### Mice and human blood samples

B6C3F1/Crl and B6D2F1/Crl female mice were purchased from Charles River Laboratories and were 10-12 weeks old at the time of transplant. Six to eight weeks old mice C57BL/6J mice were purchased from Jackson Laboratories. Mice were maintained and treated in accordance with NIH Animal Care and Use Committee (ACUC) guidelines and all animal studies were approved under the animal Study Protocol # NEI-655; EY000350-21. All animal experiments performed in this study were conducted in compliance with Institutional Animal Care and Use Committee (IACUC) guidelines. Mice were monitored daily for clinical symptoms, and body weight was measured every other day throughout the study period. Weight loss of 20% or greater is not uncommon following irradiation; however, more than 90% of transplanted mice generally recover their weight within 14 days post-irradiation. Weight loss was closely monitored, and any animal showing persistent weight loss (>20% of initial body weight) or severe clinical symptoms was promptly euthanized to minimize suffering. Prior to experiments, mice were acclimatized at least one week. Whole blood was obtained from healthy human subjects from the National Institutes of Health Department of Transfusion Medicine. Written, informed consent was obtained from all human blood donors. Supplementary Table S1 contains list and catalogue number of key reagents used for the reported experiments.

### Graft Versus Host Disease (GVHD) model

The mouse acute GVHD model was as previously described with minor modifications 24. The haploidentical B6C3F1 > B6D2F1 acute GVHD (aGVHD) model was selected because it replicates several key immunopathological features of GVHD, including chronic inflammation and immune dysregulation. While this model does not fully reproduce all aspects of human cGVHD, it provides a robust platform for investigating whether IL-35 producing B cells (i35-Bregs) can suppress GVHD. Briefly, B6D2F1/Crl (H2k^d+^H2k^b+^) recipient mice were irradiated to 10.4 Gy in a single fraction using a Gammacell 40 Cesium-137 irradiator and were randomly assigned into three groups. Transplantation was performed within 6 to 12 h and the animal were given levofloxacin-treated water (Akorn) from day 0 to 14 post-transplantation. BM single cell suspension was collected from tibias and femurs of B6C3F1/Crl (H2K^k+^H2K^b+^) donor mice and resuspended at 20×10^6^ cells/mL, then T-cell-depleted (TCD) using anti-Thy1.2 antibody (BioXCell) followed by treatment with guinea pig complement (Cedarlane). Grafts were resuspended in a total volume of 400 µl incomplete RPMI 1640 (Gibco Laboratories) and injected intravenously (i.v) via tail vein with 10×10^6^ T-cell-depleted bone marrow cells (TCD BM) plus splenocytes (50×10^6^) in the presence or absence of 1.5×10^6^ IL-35-producing CD138^+^ donor B cells. The control group were administered only 10×10^6^ T-cell-depleted bone marrow cells (TCD BM). For i35 exosome immunotherapy, GVHD was induced and each mouse in the treatment group received intravenous injection of 2×10^10^ i35 exosomes on day 0, 3, 6, 9, 12 post-transplantations. GVHD disease was blindly scored with body weight change, posture, activity, fur texture, and skin integrity as described 27, 65. Mortality/survival was assessed by Simple Survival Analysis (Kaplan Meier), Log-rank (Mantel-Cox) test and Gehan-Breslow-Wilcoxon test.

### Isolation and preparation of i35-Bregs

For producing i35 Bregs used for treatment of GVHD mice, CD19^+^ B cells were isolated from the spleen of donor B6C3F1/Cr1 mice using CD19 specific magnetic microbeads (Miltenyi Biotec, San Diego, CA). The CD19^+^B220^+^ cells were seeded at a density of 2×10^6^ cells/mL, stimulated with LPS (5 µg/mL) or anti-IgM (5 µg/mL) with anti-CD40 (5 µg/mL) and rIL-35 (50 ng/mL) at a concentration of 2×10^6^ cells/mL for 72 h. The cells were enriched for CD138-expressing B cells using CD138 antibody and sorted on FACS Aria or with Mouse CD138 microbeads from CD138^+^ plasma cell isolation kit (Miltenyi Biotec, San Diego, CA). The cells were then rested for 6 h and maintained in culture media supplemented with BAFF (10 ng/mL) and APRIL (10 ng/mL). The i35 Breg samples were evaluated by FACS and only samples enriched at up to 80% IL-35^+^ were used for both i35-Breg treatment and exosome production.

### Flow cytometry

For immunophenotyping, spleen, liver, lung, and colon from recipient mice were harvested and homogenized using gentleMACS dissociator (Miltenyi Biotec) with collagenase D digestion and lymphocytes were isolated following collagenase D digestion and percoll gradient centrifugation. Cells (3×10^6^) were stained with anti-mouse antibodies against H2K^k^ (clone AF3-12.1, BD bioscience), CD4 (clone RM4-5, BD bioscience), CD8 (clone 53-6.7, BD bioscience), CD19 (clone 1D3, BD bioscience), LAG3 (clone C9B7W, BD bioscience), PD-1 (clone J43, BD bioscience) or CTLA-4 (clone UC10-4F10-11, BD bioscience). Live/dead fixable dead cell staining kits (Invitrogen) were used to exclude dead cells. For intracellular cytokine staining, cells were stimulated for 4 h with PMA (20 ng/mL), ionomycin (1 µg/mL), and brefeldin A (Golgi Plug, BD Pharmingen). Intracellular cytokine staining was performed using BD Biosciences Cytofix/Cytoperm kit (BD Pharmingen) according to the manufacturer's instructions and stained with anti-mouse IFN-γ (clone XMG1.2, BD bioscience), IL-10 (clone JES5-16E3, BD bioscience), IL-17A (clone TC11-18H10, BD bioscience), Ki-67 (clone 16A8, BioLegend), Foxp3 (clone FJK-16s, eBioscience), p35 (clone 27537, R&D), or Ebi3 (clone 355022, R&D). For in-vitro cultured B-cells, 2×10^6^ cells were harvested and stained for surface markers using anti-mouse CD19, LAG3, PD-1 and CTLA-4, followed by intracellular cytokine staining using BD Biosciences Cytofix/Cytoperm kit (BD Pharmingen) and anti-mouse IL-12p35, EBI3 and IL-10. FACS analysis was performed on a Cytoflex analyzer (Beckman Coulter, Brea, CA) using protein-specific monoclonal antibodies and corresponding isotype control Abs (BD Pharmingen, San Diego, CA). FACS data was analyzed using FlowJo version 10.6.1. The cells were further clustered using FlowSOM and visualized using t-SNE plot in R-studio.

### Serum cytokine analysis

Mouse serum cytokine levels were measured by LEGENDplex mouse inflammation panel assays (Biolegend, CA) according to the manufacturer's instructions and read on Cytoflex analyzer (Beckman Coulter, Brea, CA). Data analysis was performed on cloud based LEGENDplex Data Analysis Software version 8.

### Histopathological analysis

Representative samples of liver, colon, lungs and eyeballs were obtained from transplanted recipients 9 days and 21 days after transplant. Each tissue was immediately immersed in 10% neutral-buffered formalin (Sigma-Aldrich), fixed for 48 h, and washed with 70% ethanol. Samples were then embedded in paraffin, cut into 5 µm thick sections, and stained with H&E. The tissue sections were examined under a microscope, and the severity of the tissue damage was graded with a semiquantitative scoring system (magnification ×200 and scale bar 200 µm). The histopathology score was graded as 0 to 4 calculated on the basis of inflammation and cell infiltration in liver, lung, colon, and spleen and analyzed 24, 83 by a pathologist who was blinded to this study. The liver was assessed for periductal lymphocyte infiltration (0, none; 1, minimal; 2, mild; 3, moderate and single-cell hepatocellular necrosis; 4, moderate to severe, single-cell hepatocellular necrosis, and portal zones with lymphocyte infiltrates). Colon was assessed for numbers of infiltrating lymphocytes into crypt epithelium and crypt hyperplasia (0, none; 1, minimal; 2, mild to moderate; 3, moderate; 4, most crypts degeneration, moderate, and mucosal ulceration). Lung was assessed for numbers of lymphocyte infiltration in perivascular cuffing (0, none; 1, 1-2 cells; 2, 2-3 cells and infiltration into parenchyma proper; 3, 4-5 cells and infiltration into parenchyma proper; 4, perivascular cuffing, >8 cells, infiltration into parenchyma proper with necrotic lesions). However, no evidence of bronchiolitis obliterans (BO)-like fibrosis, a hallmark of human cGVHD, was observed in the lung histology. The spleen cores were based on different features of the lesions counting the incidence of the red and white pulp, T zone lymphocytes, and follicles (0, rare; 1, minimal decrease; 2, mild decrease; 3, moderate decrease; 4, marked decreased). The corneal epithelial thickness was evaluated on Zeiss Zen 3.2 (Blue Edition) software, taking multiple measurements and the average plotted for each mouse.

### RNA-seq and identification of DEGs

Total RNA was isolated from human/mouse IL-35-producing B cells using RNeasy plus mini kit (Qiagen). The RNA integrity value (RIN) of the samples ranged between 9-10. For RNA-Seq, mRNA was isolated by oligo-dT beads, and a library was prepared using the standard Illumina library protocol (kit: RS-122-2101 TruSeq Stranded mRNA LT Sample prep kit). The libraries were sequenced on the NovaSeq 6000 system. Differentially expressed genes and corresponding adjusted *p-*values were determined using DEseq2 84. The significant expression patterns of differentially expressed transcripts which are satisfied with |fold change|≥2 and independent t-test raw p <0.05). We used fragments per kilobase of transcript per million mapped reads (FPKM) as a measure that should be invariant to the length of genomic features in RNA-seq. FPKM were calculated using DEseq2 84. Note: transcript counts obtained using StringTie were filtered for low quality and normalized using edgeR's TMM method. Group comparisons were done on the normalized data, using edgeR's exactTest method. A fold change of >=2 and a p-value of < 0.05 was used to select significantly differentially expressed genes (DEG) in human (4,885 DEG) and mouse (3,305 DEG). The analysis included a minimum of 139 million reads for Non-i35-Breg and 134 million reads for i35-Breg in mice; 127 million reads for Non-i35-Breg and 17 billion reads for i35-Breg in humans. In addition, differentially expressed genes were compared pairwise and visualized by volcano plots using R version 4.0.5 and the EnhancedVolcano package.

### Isolation and culture of i35 Bregs from human PBMC

PBMC was isolated by Ficoll-Paque density gradient centrifugation using the Lymphocyte Separation Medium (Corning, VA, USA). B cells were isolated from single cell suspension of PBMC using Human B-cell isolation kit (Miltenyi Biotec, Cat# 130-091-151) and were stimulated with 2 µM human CpG and 5 ug/mL anti-CD40 with 50 ng/mL rIL-35 for 72 h.

### Isolation and culture of mouse i35 Bregs

For the FACS assay and tSNE plots to show i35 Breg expression of inhibitory receptors, CD19^+^ B cells were isolated from the spleen of C57BL/6J mice using CD19 specific magnetic microbeads (Miltenyi Biotec, San Diego, CA). The CD19^+^ (2×10^6^ cells/mL) cells were stimulated with LPS (5 µg/mL) with anti-CD40 (5 µg/mL) with 50 ng/mL rIL-35 for 48 h. The cells were then washed with complete media, rested for 2 h and maintained for another 48 h in culture media supplemented with BAFF (10 ng/mL) and APRIL (10 ng/mL). At each time point, 2×10^6^ cells were harvested and subjected to Flow Cytometry and tSNE analysis as earlier described.

### i35 exosome isolation

Supernatants from in vitro mouse i35-Bregs cultures were subjected to sequential centrifugations at 300 xg (10 min), 3000 xg (10 min) and 12000 xg (10 min) and exosomes were isolated using the ExoQuick-ULTRA kit as previously described 62. Note that exosome production was performed with culture media supplemented with Exosome depleted FBS (System Biosciencesm Cat# EXO-FBS-250A-1). For i35-exosome isolation by fractionation of i35-Bregs supernatant, the cleared supernatant post 12000 xg (10 min) centrifugation was further subjected to ultracentrifugation at 100,000 xg (2 h) followed by washing of the particulate fraction in PBS at 100,000 xg (2 h). Exosome size distribution was measured by Nanoparticle Tracking Analysis using the NanoSight system (Malvern Panalytical, MA, USA) and Tunable Resistive Pulse Sensing (TRPS) using the EXOID system. Size range of the purified exosomes was established by electron microscopy. Briefly, serial dilution of purified exosomes was prepared in Eppendorf tubes. For the whole mount, aliquots of 5 µL from each fraction was dropped on a formvar carbon-coated mesh grid (Electron Microscopy Sciences, Hatfield, PA) and air dried overnight before staining with uranyl acetate and viewed in a JEOL JEM 1010 transmission electron microscope. Expression of exosome markers or IL-35 subunit proteins were characterized and validated by Western blotting and validation that i35-exosomes are indeed exosomes based on criteria established by the International Society for extracellular vesicles (ISEV). Additional description of methods, reagents and instrumentation used for sample collection, EV separation and quantification are also provided as Supplementary data (Supplementary Figure S7 and Table S1).

### Immunofluorescence assay and proximity ligation assay

B cells were activated as earlier described and adhered to glass slides. Briefly, the cells were fixed with BD-Fixation/permeabilization kit, adhered to glass slides by the Cytospin method or incubated for 30 min on Poly-L-lysine coated slide. Cells were blocked in 10% goat serum, 1% BSA, 0.1% Triton for 1 h and in MOM blocking solution (Invitrogen) if the primary antibody is of mouse origin. Cells were then incubated with rabbit anti-CD81 (Cell Signaling), mouse anti-Ebi3 and rat anti-p35 (eBioscience). Cells were washed 3 time for 10 min each and incubated with Alexa 488 (anti-rat), Alexa 568 (anti-rabbit) and 647 (anti-mouse) secondary antibodies. For the proximity ligation assay, slides were processed and fixed as described above, followed by blocking, primary antibody incubation with Rabbit anti-CD81 (Cell signaling) + Mouse anti-p35/Ebi3 (Santa Cruz) antibody incubation, probe incubation, ligation and polymerase amplification using the Duolink PLA Fluorescence kit (Millipore Sigma) following manufacturer's instructions. Slides were mounted using Vectashield mounting medium with DAPI/Vectashield Vibrant Hardset mounting media with DAPI staining and images acquired using Zeiss700 / Zeiss 880 confocal microscope with Airyscan.

### Protein complex modeling

AlphaFold-Multimer 85, 86 was used to predict the protein complexes. Protein sequences without the signal peptide were used as input for the AlphaFold-Multimer predictions. Top scoring model was relaxed and refined to obtain the final protein complex structure. ChimeraX 87 was used to visualize and analyze the predicted complex structures.

### Statistical analysis

Graphpad Prism 9 was used for graphing and statistical analyses. Survival distributions were compared using Simple Survival Analysis (Kaplan Meier), the Log-rank (Mantel-Cox) and Gehan-Breslow-Wilcoxon tests. One-way ANOVA with Dunnett's or Holm-Sidak post hoc test correction was used for multi-group analysis. Pairwise analysis was performed using student t-test for normally distributed data, otherwise Mann-Whitney test were performed. A p-value of < 0.05 was considered statistically significant. Data are depicted as mean ± SEM.

## Supplementary Material

Supplementary figures and table.

## Abbreviations

allo-HSCT: Allogeneic hematopoietic stem cell transplantation
GVHD: graft-versus-host disease
aGVHD: acute graft-versus-host disease
cGVHD: chronic graft-versus-host disease
IL: interleukin
Ebi3: Epstein-Barr virus induced gene 3
Breg: regulatory B cell
Treg: regulatory T cell
i35-Bregs: IL-35-producing B cells
TCD: T-cell-depleted
TCD BM: T-cell-depleted bone marrow cells
FACS: fluorescence-activated cell sorting
BCR: B cell receptor
BAFF: B-cell activating factor
APRIL: a proliferation-inducing ligand
PD-1: programmed cell death protein 1
LAG-3: lymphocyte-activation gene 3
CTLA-4: cytotoxic T-lymphocyte associated protein 4
MHC: major histocompatibility complex
IFNγ: interferon gamma
TNF: tumor necrosis
MCP-1: monocyte chemoattractant protein-1
IL-1α: interleukin 1 alpha
IL-1β: interleukin 1 beta
IL-10: interleukin 10
IL-12: interleukin 12
IL-17: interleukin 17
IL-35: interleukin 35
DP: double positive
LN: lymph node
DEG: monocyte chemoattractant protein-1
FPKM: fragments per kilobase of transcript per million mapped reads
TMM: trimmed mean of M-values
PBMC: peripheral blood mononuclear cells
CpG: cytosine-phosphate-guanine (CpG motifs are DNA sequences)
TRPS: Tunable Resistive Pulse Sensing
ISEV: International Society for extracellular vesicles
EV: extracellular vesicles
PLA: proximity ligation assay
