# Supplementary material for: Interleukin 35-producing B cells prolong the survival of GVHD mice by secreting exosomes with membrane-bound IL-35 and upregulating PD-1/LAG-3 checkpoint proteins
Source: Theranostics. 2025 Feb 25;15(8):3610–26. doi: 10.7150/thno.105069 (PMC11905137; doi:10.7150/thno.105069)
Supplement: Supplementary file 1 — Supplementary figures and table. [file thnov15p3610s1.pdf]

Figure S1A

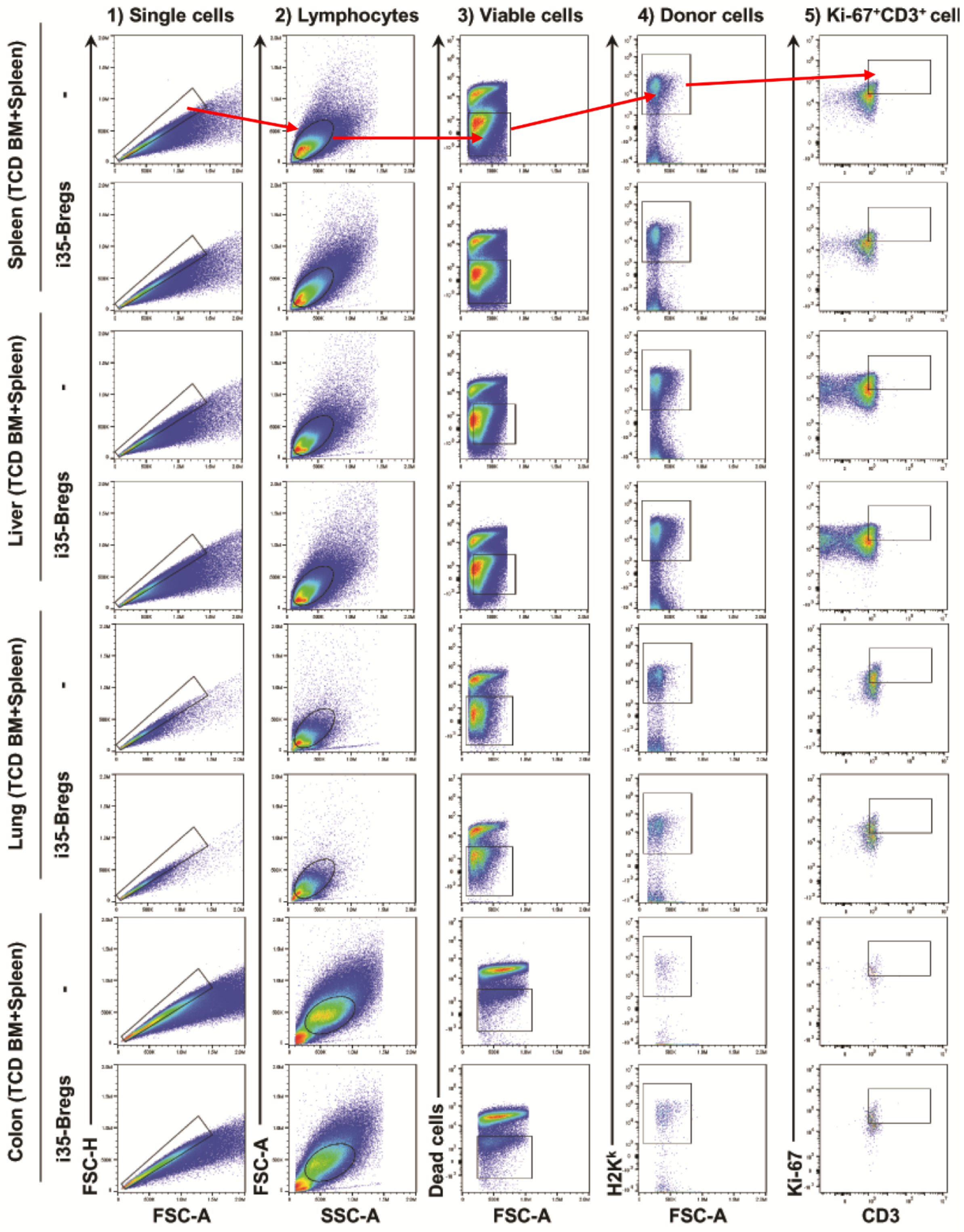

1) Single cells
2) Lymphocytes
3) Viable cells
4) Donor cells
5) Ki-67⁺CD3⁺ cell
Spleen (TCD BM+Spleen)
Liver (TCD BM+Spleen)
Lung (TCD BM+Spleen)
Colon (TCD BM+Spleen)
i35-Bregs
i35-Bregs
i35-Bregs
i35-Bregs
FSC-H
FSC-A
FSC-A
SSC-A
Dead cells
FSC-A
H2Kᵏ
FSC-A
Ki-67
CD3

Figure S1B

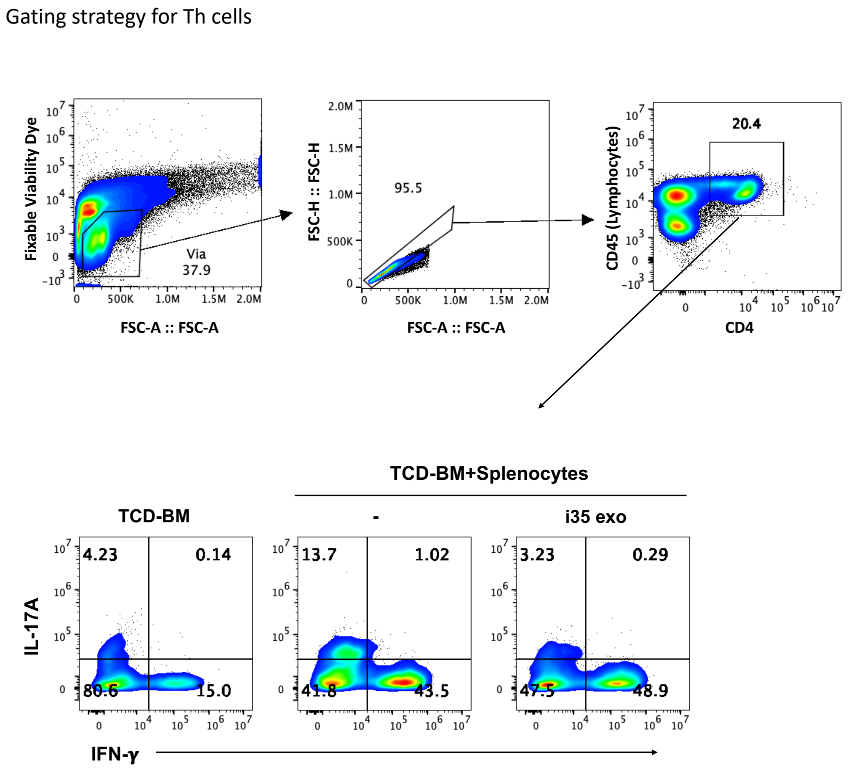

Figure S1. Gating strategy for T cells. Cells were isolated from the spleen, liver, lung, lymph node and colon on day-9 and day-21 post-transplantation and $CD4^{+}$ T cells or $CD8^{+}$ T cells were analyzed by Flow cytometry. **(A)** The gating strategy for proliferation (Ki-67^{+}) of donor ($H2k^{k+}$) $CD3^{+}$ T cells is depicted, with representative flow cytometry plots day-9 shown. **(B)** Representative flow cytometry plot for evaluating IFN-γ and IL-17A expression in $CD4^{+}$ T cells.

Figure S2

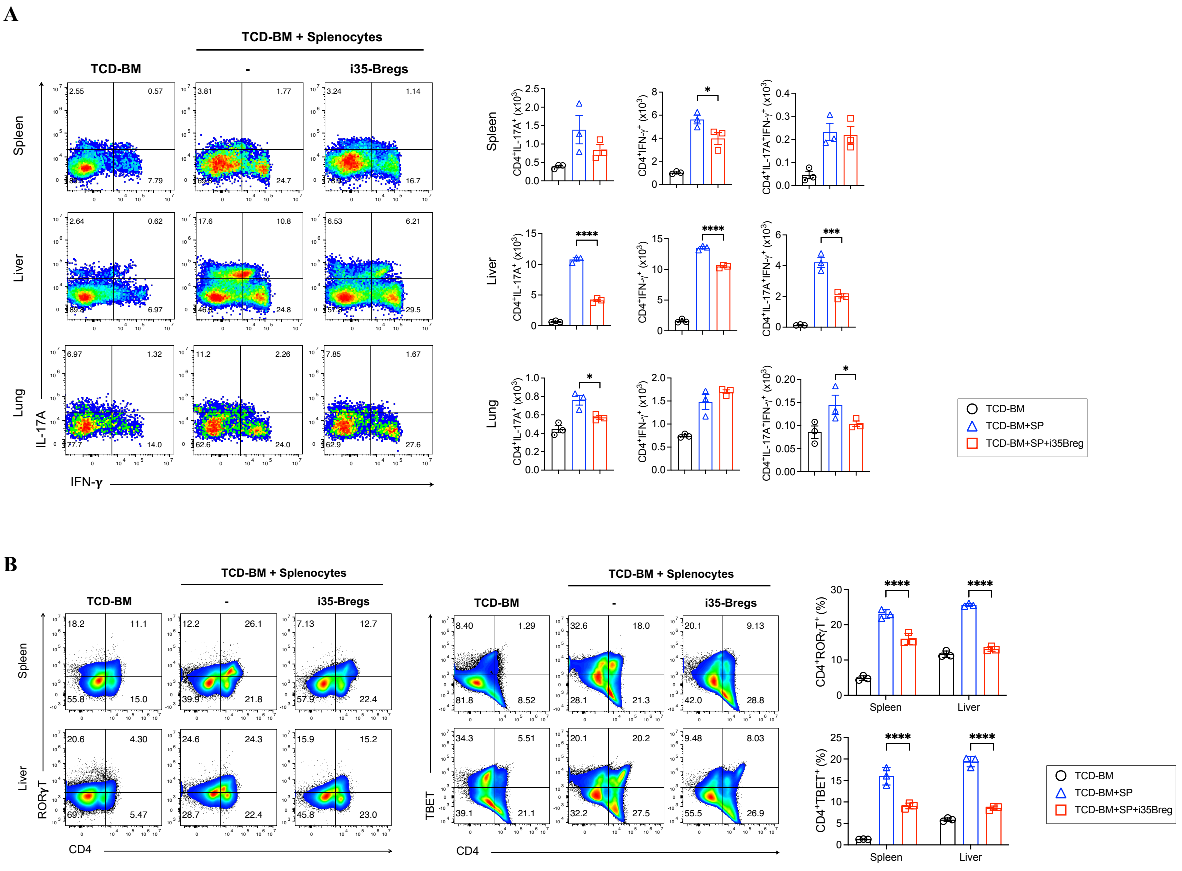

Figure S2. i35-Breg cells inhibited alloreactive T cells at day-21. Cells were isolated from the spleen and liver on day-21 post-transplantation and CD4$^+$ T cells were analyzed by intracellular cytokine staining assay. (A) Proportion of CD4$^+$ T cells expressing IL-17 and/or IFN-γ in the spleen, liver or lungs at Day 21. The quadrants show proportion of cells while statistics show cell number. (B) Proportion of CD4$^+$ T cells expressing RORγT and T-bet. Data is representative from three independent experiments.

Figure S3

Figure S3A

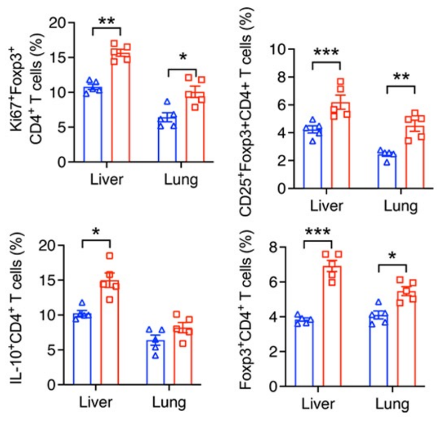

Figure S3B

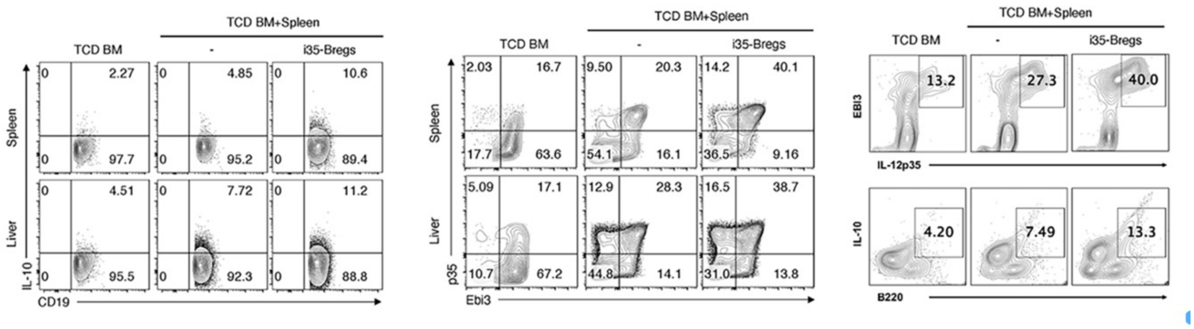

Figure S3. i35-Breg cells induced expansion of Treg and Bregs. (A) Cells were isolated from the spleen and liver on day-9 post-transplantation and CD4$^+$ T cells were analyzed by intracellular cytokine staining assay. Numbers in quadrants indicate percentage of proliferating CD4$^+$ T cells expressing Foxp3 and/or CD25 **or** IL-10**.** Bars show percent Ki-67$^+$Foxp3$^+$, Foxp3$^+$CD25$^+$, or IL-10-expressing Foxp3$^+$ CD4$^+$ T. **(B)** CD19$^+$ and B220$^+$ B cells in the liver or spleen were analyzed by intracellular cytokine staining assay and representative flow cytometry plots are shown. Numbers in quadrants indicate percentage of B cells expressing IL-10, IL-35 (p35$^+$Ebi3$^+$), IL-35 or IL-10. Data represented from three independent experiments.

Supplementary Figure S4

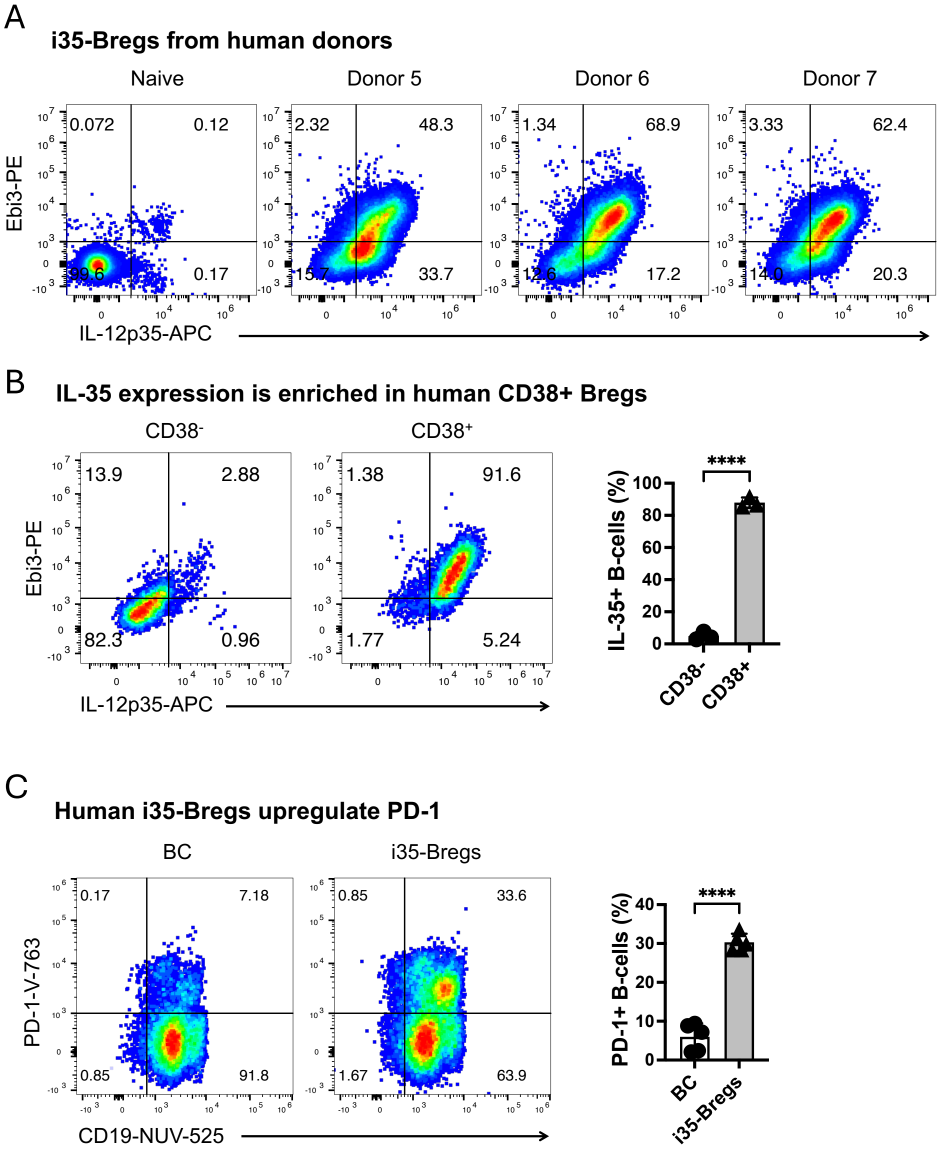

Supplementary Figure S4. Human i35-Bregs express high levels of immune checkpoint proteins. PBMC was collected from blood donors and CD19+ B cells were isolated from the human donor PBMC. The B cells were cultured in the presence of anti-IgM and anti-CD40. **(A)** IL-35 expressing Bregs from 3 donors. **(B)** IL-35 Bregs were further enriched by sorting CD38+ cells. **(C)** PD-1 expression by human i35-Bregs compared to conventional B cells.

Figure S5

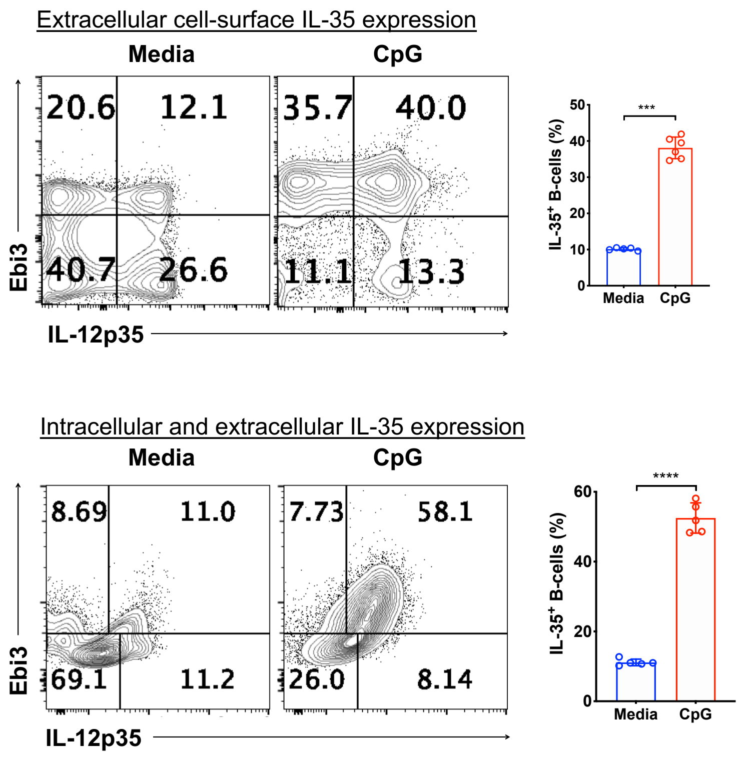

Figure S5. Detection of membrane-bound IL-35 on activated human B cells

Human CD19^{+} B cells were activated with CpG for 72 h. For detection of membrane-bound IL-35 (p35^{+} and Ebi3^{+}), the cells were analyzed by FACS without permeabilization. For detection of intracellular IL-35 expression, the cells were permeabilized and then subjected to intracellular cytokine staining assay.

Figure S6.

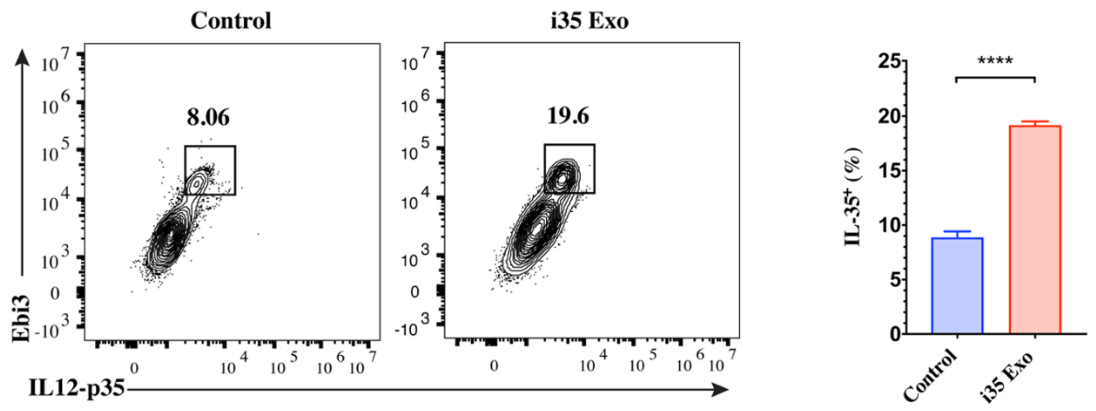

Figure S6. Uptake of i35 Exosome by bystander conventional CD19$^+$ B cells

Conventional B cells were co-cultured with i35 Exosomes or Control Exosomes at 2 x 10^{10} particles/mL complete RPMI supplemented with IL-15/IL-2 (10 ng/mL each). After 72 h incubation, cells were harvested and stained for surface detection of IL-35 subunits (p35 and Ebi3). Representative flow cytometry plots and graph showing percentage of IL-35 expressing B cells.

Figure S7.

ISEV-18 recommendation Checklist

EV nomenclature:

The Extracellular vesicles used for this study, was of endosomal origin and extracted from cell culture supernatant (culture density: $2x10^6$ cells/mL) and are of size range >50 nm to <150 nm with mean size ~100 nm, which we thus referred to as exosomes in this publication. Further, the culture media used for experiments was supplemented with exosome depleted FBS (System Biosciencesm Cat# EXO-FBS-250A-1) to prevent contamination from ectosomal sources.

Sample collection and pre-processing:

Supernatants from in vitro mouse i35 Bregs cultures were subjected to sequential centrifugations at 300 xg (10 min), 3000 xg (10 min) and 12000 xg (10 min) to remove the cells, dead cells and cell debris respectively before any isolation techniques.

EV separation and concentration:

We used two methods for exosomes isolation, namely: ExoQuick-TC kit and ultracentrifugation. The exosome samples used for treating animals were isolated using the ExoQuick-TC kit with purification step as previously described. In these experiments, there was no need for further concentration of the exosomes. For samples used for Western blots, i35 exosomes were isolated by fractionation of i35 Bregs supernatant by ultracentrifugation. The cleared supernatant post 12000xg (10 min) centrifugation was further subjected to ultracentrifugation at 100,000 xg (2 h) followed by washing of the particulate fraction in PBS at 100,000 xg (2 h).

Characterization:

a. **Size concentration distribution by NTA**

We measured exosome size distribution by Nanoparticle Tracking Analysis using the NanoSight system (Malvern Panalytical, MA, USA) and the expression of exosome markers (CD81, see Figure 5A-B). The NTA size and concentration plot for the sample used for treating animals is attached below:

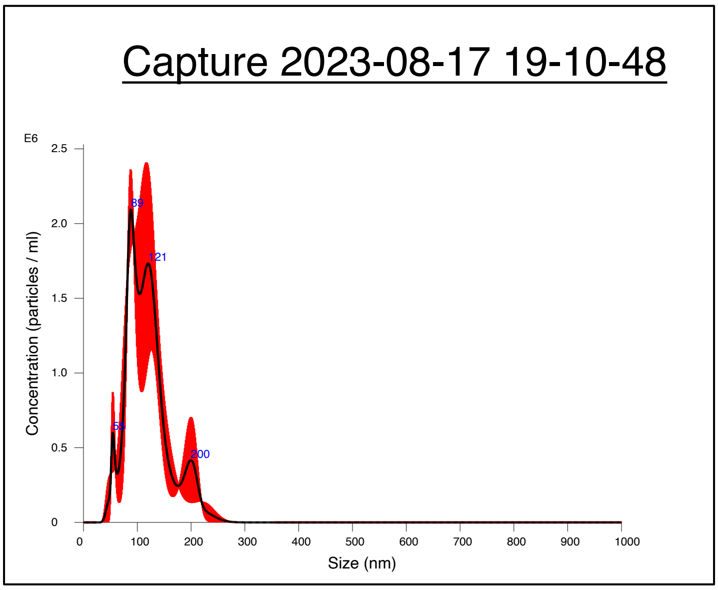

Summary of NTA results are as follows:

Stats: Merged Data

Mean: 118.3 nm

Mode: 88.2 nm

SD: 39.4 nm

D10: 76.1 nm

D50: 111.7 nm

D90: 178.6 nm

b. **Size and concentration measurement by Exoid (Izon)**

The samples used for other in vitro assays were further evaluated for size and concentration on

the Exoid instrument (CS 1.0, Izon). The result of Exoid measurement is as follows:

Analysis Report

Sample: **2021-08-18 Soln S_P2**
Measurement date: Wednesday, August 18, 2021 1:48 PM

Sample Statistics

Particle diameter (nm)

Mean:	114 (Std Dev=42.7)	d50:	98		
Mode:	82	d10:	82	d90:	171
Maximum:	360	d90/d10:	2.09		
Minimum:	79	Span:	0.91		

Concentration [0-∞] (particles/ml)

Measured Concentration: 1.00e+09
Raw Concentration: 1.00e+10

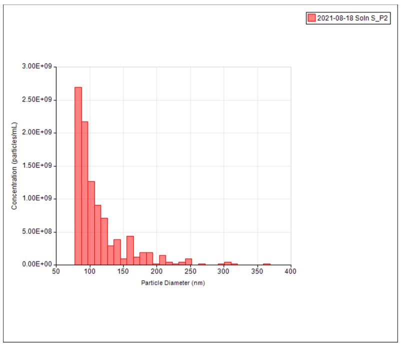

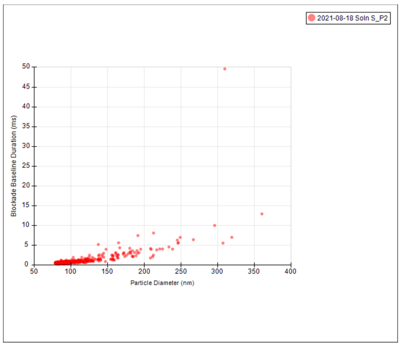

Measurement Details

Software version: 1.0.2.9
Capture assistant: None   Gold Standard Analysis: no
Investigation: 2021-08-18 Intro
Sample ID: 2021-08-18 Soln S_P2
db977e4a-ae48-4d0e-920a-9eefd6862eb9
Electrolyte: PBS (137 mM)
Dilution: 10
Pressure: 1000
Nanopore ID: A73357
Nanopore Type: N/A
Calibration sample ID(s):TKP200_P2; TKP200_P3
Notes:

c. Sample evaluation by electron microscopy

To confirm the purity of the exosome fraction and further evaluate the size by imaging, further

analysis for size measurement was performed on an aliquot of the exosomes samples using electron microscopy as below:

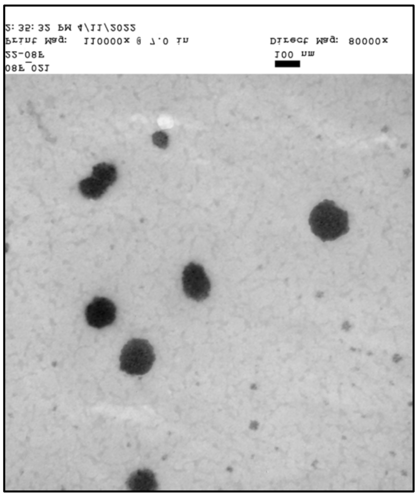

80000x magnification with measurement.

Figure S8

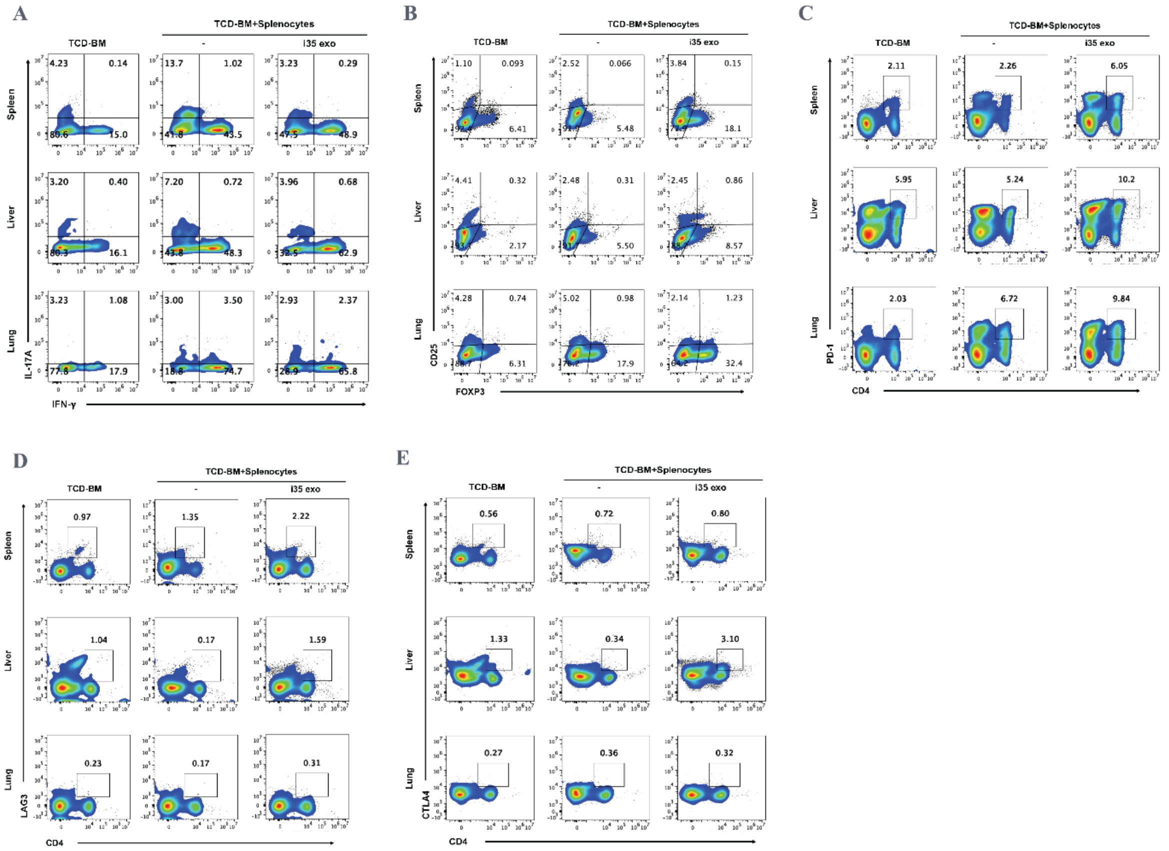

Figure S8. Adoptive transfer of IL-35-containing exosomes (i35-Exosomes) suppressed GVHD. GVHD was induced in irradiated B6D2F1/J ($H2K^{d+}$) mice by transplanting $10{\times}10^6$ TCD-BM cells and/or TCD-BM plus $50{\times}10^6$ spleen cells (TCD-BM+SP) from donor B6C3F1/J ($H2K^{k+}$) mice. The treatment group were treated similarly with TCD-BM+SP (n = 5) but also received $2{\times}10^{10}$ i35-exosomes/mouse (TCD-BM+SP+i35-exo) intravenous injection (n = 5) on day 0, 3, 6, 9, 12 after transplantation. Cells isolated from the spleen, liver or lung were analyzed by intracellular cytokine assay and/or cell surface FACS. **(A)** Representative flow cytometry plots of $CD4^+$ T cells expressing IL-17A$^+$ and/or IFN-γ^+ **(A)**, Foxp3$^+$ and/or CD25$^+$ **(B)**, PD-1$^+$ **(C)**, LAG3$^+$ **(D)** or CTLA-4$^+$ **(E)**. Data represent three independent experiments.

SUPPLEMENTARY TABLE 1

REAGENT or RESOURCE	SOURCE	IDENTIFIER
**Antibodies**		
Anti-CD138	BD Biosciences	Cat# 561070, RRID:AB_2033998
Anti-Thy1.2	Bioxcell	Cat# BE0066, RRID:AB_1107682
$H2K^k$ (clone AF3-12.1)	BD Biosciences	Cat# 750225, RRID:AB_2874424
Anti-CD4 (clone RM4-5)	BD Biosciences	Cat# 550954, RRID:AB_393977
Anti-CD8 (clone 53-6.7)	R and D Systems	Cat# FAB116P, RRID:AB_356999
Anti-CD19 (clone 1D3)	BD Biosciences	Cat# 557655, RRID:AB_396770
Anti-LAG3 (clone C9B7W)	BD Biosciences	Cat# 741594, RRID:AB_2871003
Anti-PD-1 (clone J43)	BD Biosciences	Cat# 749422, RRID:AB_2873791
Anti-CTLA4 (clone UC10-4F10-11)	BD Biosciences	Cat# 564332, RRID:AB_2732917
Anti-IFN-g (clone XMG1.2)	BD Biosciences	Cat# 554413, RRID:AB_398551
Anti-IL-10 (clone JES5-16E3)	BD Biosciences	Cat# 554466, RRID:AB_395411
Anti-IL-17A (clone TC11-18H10)	BD Biosciences	Cat# 560666, RRID:AB_1937311
Anti-Ki-67 (clone 16A8)	BioLegend	Cat# 652419, RRID:AB_2564284
Anti-Foxp3 (clone FJK-16s)	eBioscience	Cat# 25-5773-82, RRID:AB_891552
Anti-IL-12p35 (clone 27537)	R and D Systems	Cat# IC2191A, RRID:AB_1964616
Anti-Ebi3 (clone 355022)	R and D Systems	Cat# IC18341C, RRID:AB_3097736
Anti-CD81 (DSO2Q)	Cell Signaling Tec hnology	Cat# 10037, RRID:AB_2714207
Anti-Ebi3	eBioscience	Cat# 14-7273-80, RRID:AB_1659711
Anti-IL-12p35	eBioscience	Cat# 14-7122-85, RRID:AB_468443
CD19 MicroBeads, mouse	Miltenyi Biotec	Cat# 130-121-301, RRID:AB_2827612
CD19 MicroBeads, human	Miltenyi Biotec	Cat# 130-050-301, RRID:AB_2848166
AffiniPure™ $F(ab')_2$ Fragment Goat Anti-Mouse IgM, μ chain specific	Jackson Immuno-R esearch Lab.	Cat# 115-006-020, RRID:AB_2338469
InVivoMAb anti-mouse CD40	Bioxcell	Cat# BE0016-2, RRID:AB_1107647
**Chemicals, Peptides, and Recombinant Proteins**		
Guinea pig complement	Cedarlane	Cat# CL5000-R
Levofloxacin	Akorn	Cat# 17478-107-30
Recombinant Mouse BAFF/BLyS/TN FSF13B Protein	R&D Systems	Cat# 8876-BF
Recombinant Mouse APRIL/TNFSF1 3 Protein	R&D Systems	Cat# 7907-AP
Lipopolysaccharides from *Escherichia coli* O111:B4	Sigma-Aldrich	Cat# L2630-100MG
BD GolgiPlug	BD	Cat# 555029
**Critical commercial Assays**		

Duolink In Situ PLA Probe Anti-Rabbit PLUS antibody,	Sigma-Aldrich	Cat# DUO92002, RRID:AB_2810940
Duolink In Situ PLA Probe Anti-Mouse MINUS Antibody	Sigma-Aldrich	Cat# DUO92004, RRID:AB_2713942
ExoQuick-TC kit	System Biosciences	Cat# EXOTC50A-1
LEGENDplex™ Mouse Inflammation Panel (13-plex)	Biolegend	Cat# 740150
**Experimental Models: Organisms/Strains**		
B6C3F1/Crl	Charles River Laboratory	RRID:IMSR_CRL:031
B6D2F1/Crl	Charles River Laboratory	RRID:IMSR_CRL:099
C57BL/6J	Jackson Laboratory	RRID:MGI:3028467
**Deposited data**		
Raw and processed RNA-seq data	This Paper	NCBI GEO: GSE263242
**Software and algorithms**		
AlphaFold Multimer	Google DeepMind	https://deepmind.google/technologies/alphafold/
ChimeraX-1.7	UCSF	https://www.rbvi.ucsf.edu/chimerax/
ZEN Blue	Zeiss	ZEN version 3.8
Prism 9	GraphPad Software, LLC.	Prism version 9.5.1
FlowJo	BD	FlowJo version 10.9.0
Imaris	Oxford Instruments	Imaris version 10.1
Other		
Gammacell 40 Cesium-137 irradiator	Best Theratronics	NA
Zeiss LSM 880 Airy scan	Zeiss	NA
Axio Observer 7 Microscope	Zeiss	NA
NIH HPC Biowulf cluster	NIH Biowulf	https://hpc.nih.gov
